# Effectiveness of Workplace-Based Diet and Lifestyle Interventions on Risk Factors in Workers with Metabolic Syndrome: A Systematic Review, Meta-Analysis and Meta-Regression

**DOI:** 10.3390/nu13124560

**Published:** 2021-12-20

**Authors:** Alicia Gea Cabrera, Pablo Caballero, Carmina Wanden-Berghe, María Sanz-Lorente, Elsa López-Pintor

**Affiliations:** 1 Department of Engineering, Area of Pharmacy and Pharmaceutical Technology, Universidad Miguel Hernández, 03550 Alicante, Spain; ali_ftca@hotmail.com; 2 Department of Community Nursing, Preventive Medicine and Public Health and History of Science (Spain), University of Alicante, San Vicente del Raspeig, 03690 Alicante, Spain; pablo.caballero@ua.es; 3 Grupo de Nutrición Clínica y HAD del Instituto de Investigación Sanitaria y Biomédica de Alicante (ISABIAL-Fundación FISABIO), Hospital General Universitario de Alicante, 03010 Alicante, Spain; carminaw@telefonica.net; 4 Department of Public Health and History of Science, Universidad Miguel Hernández, 03550 Alicante, Spain; msanzlor@gmail.com; 5 CIBER of Epidemiology and Public Health, CIBERESP, 28029 Madrid, Spain

**Keywords:** metabolic syndrome, occupational health, workplace, diet, food and nutrition, systematic review

## Abstract

Workplace health interventions are essential to improve the health and well-being of workers and promote healthy lifestyle behaviours. We carried out a systematic review, meta-analysis and meta-regression of articles measuring the association between workplace dietary interventions and MetS risk. We recovered potentially eligible studies by searching MEDLINE, the Cochrane Library, Embase, Scopus and Web of Science, using the terms “Metabolic syndrome” and “Occupational Health”. A total of 311 references were retrieved and 13 documents were selected after applying the inclusion and exclusion criteria. Dietary interventions were grouped into six main types: basic education/counselling; specific diet/changes in diet and food intake; behavioural change/coaching; physical exercise; stress management; and internet/social networks. Most programmes included several components. The interventions considered together are beneficial, but the clinical results reflect only a minimal impact on MetS risk. According to the metaregression, the interventions with the greatest impact were those that used coaching techniques and those that promoted physical activity, leading to increased HDL (effect size = 1.58, sig = 0.043; and 2.02, 0.015, respectively) and decreased BMI (effect size = −0.79, sig = −0.009; and −0.77, 0.034, respectively). In contrast, interventions offering information on healthy habits and lifestyle had the contrary effect, leading to increased BMI (effect size = 0.78, sig = 0.006), systolic blood pressure (effect size = 4.85, sig = 0.038) and diastolic blood pressure (effect size = 3.34, sig = 0.001). It is necessary to improve the efficiency of dietary interventions aimed at lowering MetS risk in workers.

## 1. Introduction

Metabolic syndrome (MetS) constitutes a major public health problem, not only because of its increasing prevalence—currently estimated at 25% worldwide [1]—but also because of its clinical, economic and humanistic impact.

The WHO defines MetS as a pathological condition characterised by abdominal obesity, insulin resistance, hypertension and hyperlipidaemia. Slightly different definitions have been provided for this syndrome. The harmonized definition of the International Diabetes Federation Task Force on Epidemiology and Prevention; National Heart, Lung, and Blood Institute; American Heart Association; World Heart Federation; International Atherosclerosis Society; and International Association for the Study of Obesity [2] defined MetS as the presence of three or more of the following five risk factors for cardiovascular disease (CVD): (1) abdominal obesity (determined by waist circumference), (2) low levels of high-density lipoprotein cholesterol (HDL-c), (3) elevated blood pressure or use of medication for blood pressure, (4) elevated fasting glucose levels or use of medication to control blood glucose, and (5) elevated triglyceride levels.

MetS is associated with a two-fold increase in risk of CVD, CVD mortality, myocardial infarction and stroke; a 1.5-fold increase in risk of all-cause mortality [3]. Multifaceted lifestyle interventions, including diet and physical exercise, are recommended as the first line treatment for MetS [4]. Weight loss has a positive impact on all MetS factors, whereas physical activity reduces insulin resistance, increases fitness, and improves energy balance, thus reducing cardiovascular disease risk [4]. A healthy lifestyle, centred on appropriate diet and nutritional habits, together with supervised or unsupervised exercise, is therefore critical for preventing or delaying the onset of MetS in susceptible individuals and preventing CVD in those with MetS [5].

With regard to dietary habits, the available evidence suggests that certain nutrients, foods and dietary patterns have a beneficial impact on MetS, resulting in improved metabolic profiles and quality of life [6]. For example, replacing carbohydrates with polyunsaturated fats lowers triglycerides (TG) increases high density lipoprotein (HDL) cholesterol, and lowers blood pressure [7]. In addition, studies have shown that consuming sugar-sweetened and alcoholic beverages can increase MetS risk [8,9].

Consequently, dietary recommendations for managing MetS include weight loss through healthy eating, a plant-based Mediterranean diet, and other specific dietary recommendations, such as limited saturated and trans fats, increased dietary fibre, reduced intake of sugar-sweetened beverages, moderate alcohol intake, and restricted salt intake [10].

The impact of MetS in the working population is a subject of serious concern, as the consequences go beyond increased cardiovascular risk [11]. Schultz et al. [12], who reported a 30.2% prevalence of MetS in factory employees, also found that the affected individuals were more likely to self-report other medical conditions such as arthritis, chronic pain or heartburn. In addition, MetS impacts on the productivity of employees and companies, both directly (medical and pharmacy costs due to illness) and indirectly (short-term disability absence costs, presenteeism) [12]. The estimated annual health costs of a worker with MetS are 3.66 times those of a healthy worker [13].

In this context, workplace health promotion programs are growing in popularity. These initiatives help companies to identify MetS risks and take measures to reduce them in order to avoid productivity losses. Several studies have assessed the impact of behavioural change interventions on diet, physical activity or weight loss in the workplace [14,15,16], but few have examined the effect of dietary interventions in isolation, and to date there is no evidence showing which interventions are most effective at changing dietary and nutritional habits in the context of a workplace programme. Consequently, it is challenging to determine, from a nutritional perspective, which interventions can most effectively prevent MetS, improve cardiometabolic health, and thus generate most value in workplace settings.

This study aims to systematically review and summarise the literature on diet, food and nutrition-based interventions and their impact on workers’ MetS risk factors. 

## 2. Materials and Methods

### 2.1. Design

Systematic review, meta-analysis and meta-regression of articles measuring the association between workplace dietary interventions and MetS risk. The systematic review was conducted in agreement with the procedures and verification list described by PRISMA Statement.

### 2.2. Source of Data

We recovered potentially eligible studies by searching the following online bibliographic databases: MEDLINE (via PubMed), the Cochrane Library, Embase, Scopus and Web of Science.

### 2.3. Search Strategy

To define the search terms we consulted the Medical Subject Headings thesaurus developed by the U.S. National Library of Medicine. The search scheme was planned in three dimensions:

- Population: adult workers aged 18 years or older.
- Intervention: dietary interventions aimed at improving MetS risk factors in workers. To ensure we recovered all the relevant information, given that interventions could include several components, we performed an open search for MetS interventions and then manually selected only those that focused on nutrition, discarding those centred exclusively on other aspects, such as physical activity, stress or sleep.
- Outcome: clinical impact of the interventions in terms of improvement in MetS risk factors and other aspects related to occupational health.

Our search syntax therefore consisted of a Boolean combination of two terms: (Metabolic syndrome) AND (Occupational Health).

#### 2.3.1. Term 1: Metabolic Syndrome

((“Metabolic Syndrome” [Mesh] OR “Metabolic Syndrome” [Title/Abstract] OR “Metabolic Syndrome X” [Title/Abstract] OR “Insulin Resistance Syndrome” [Title/Abstract] OR “Metabolic X Syndrome” [Title/Abstract] OR “Dysmetabolic Syndrome” [Title/Abstract] OR “Reaven Syndrome X” [Title/Abstract] OR “Metabolic Cardiovascular Syndrome” [Title/Abstract])).

#### 2.3.2. Term 2: Occupational Health

((“Occupational Health”[Mesh] OR “Occupational Health”[Title/Abstract] OR “Industrial Hygiene”[Title/Abstract] OR “Industrial Health”[Title/Abstract] OR “Occupational Safety”[Title/Abstract] OR “Employee Health”[Title/Abstract] OR “Occupational Exposure”[Mesh] OR “Occupational Exposure”[Title/Abstract] OR “Occupational Stress”[Mesh] OR “Occupational Stress”[Title/Abstract] OR “Occupational Diseases”[Mesh] OR “Occupational Diseases”[Title/Abstract] OR “Occupational Hazard”[Title/Abstract] OR “Occupational Medicine”[Mesh] OR “Occupational Medicine”[Title/Abstract] OR “Occupational Health Safety”[Title/Abstract] OR “Occupational Health Services”[Title/Abstract] OR “Occupational Health Services”[Mesh] OR “Occupational Stressor”[Title/Abstract] OR “Occupational Factors”[Title/Abstract] OR “Workplace”[Mesh] OR “Workplace”[Title/Abstract] OR “Workplace Health”[Title/Abstract] OR “Workplace Safety”[Title/Abstract] OR “Safety Climate”[Title/Abstract] OR “Total Worker Health”[Title/Abstract] OR “Working Environment”[Title/Abstract] OR “Job Satisfaction”[Mesh] OR “Job Satisfaction”[Title/Abstract] OR “Job Stress”[Title/Abstract] OR “Job Security”[Title/Abstract] OR “Psychosocial Working Conditions”[Title/Abstract] OR “Employee Health”[Title/Abstract] OR “Shiftwork”[Title/Abstract] OR “Work Hygiene”[Title/Abstract])).

The following filters were applied: “Humans”, “Adult 18+ years”, and “Comparative Study” or “Clinical Trial”.

The search syntax was developed for MEDLINE, then adapted to each of the remaining databases. The search was performed from the first available date until the day of the last query of the databases (initial search in MEDLINE 2 May 2020). To reduce the possibility of publication bias, we reviewed the reference lists of the selected articles and of relevant guidelines. Furthermore, experts in the domain were contacted by mail to avoid issues regarding possible grey literature (materials and research produced by organisations outside the traditional commercial or academic publishing and distribution channels).

### 2.4. Final Selection of Articles

Registries that met the following inclusion criteria were accepted for review: articles with clinical trial or comparative study design that met the search criteria and that were published in peer-reviewed journals in English, Spanish or Portuguese.

Exclusion criteria were unavailability of the complete text, lack of causal relationship between metabolic syndrome and occupational health, and interventions not focused on diet in adults.

We preselected articles by carefully reading the titles and abstracts to verify suitability according to the established inclusion criteria. We then performed a full text review of all preselected studies, excluding duplicates and articles that did not provide the relevant data.

Eligible articles were independently selected by two authors (AGC and ELP). To validate study inclusion, we established that inter-rater agreement (Cohen’s Kappa coefficient) should be greater than 60% [17]. Provided that this condition was met, any discrepancies were resolved by consulting a third author (CWB) and subsequently reaching a consensus among all authors.

### 2.5. Data Extraction

Double entry tables were used to ensure data accuracy. Where inconsistencies were detected between two entries, the original articles were consulted for verification.

#### Study Variables for Each Article

The studies were grouped by study variables to ease comprehension of the results. We extracted the following data from each article:

- Author: first author.
- Year: year of publication.
- Design: procedures, methods and techniques. We only accepted clinical trials and comparative studies.
- Country/MetS criteria: country where the intervention took place and diagnostic classification of MetS.
- Worker profile and work environment: place of work and characteristics of the adult workers who received the intervention.
- Type of intervention: description and characteristics of the dietary intervention.
- Effect: causal relationship derived from the results. We recorded the clinical and anthropometric results derived from the interventions, as well as other outcomes of interest related to improvement of MetS and/or occupational health parameters.

### 2.6. Quality of Reporting of the Selected Documents

We used the CONSORT (Consolidated Standards of Reporting Trials) statements to assess quality of reporting [18]. This checklist contains 25 essential aspects that should be described in all studies. One point was assigned for each item present (if not applicable, it was not scored). When an item was composed of several points, the points were assessed independently and then averaged to give a final score. In this way, no item could receive a score above 1 point.

### 2.7. Obsolescence

We calculated Burton–Kebler half-life (median age) and Price Index (percentage of articles less than 5 years old) to determine whether clinical trials were up-to-date or obsolete.

### 2.8. Meta-Analysis and Meta-Regression

We analysed the global effect size through a meta-analysis of the included articles, considering the following variables: waist circumference (WC), body mass index (BMI), total cholesterol (TC), high-density lipoprotein cholesterol (HDL-c), low-density lipoprotein cholesterol (LDL-c), triglycerides (TG), systolic blood pressure (SBP), diastolic blood pressure (DBP), and fasting blood glucose. For this analysis we adopted the random effects model. The effect sizes and 95% confidence intervals were presented in forest plots together with the coefficient of determination (R-squared), the Tau-squared value, and *p* value. 

To examine the influence of each study on effect size, we used the leave-one-out method, which consists of omitting one study at a time and recalculating heterogeneity [19]. We also created the scatter plot introduced by Baujat et al. [20]. In this graph, the X axis shows the contribution of each study to heterogeneity, and the Y axis shows the influence of each study on the overall effect size, with the strongest influencers located in the upper right-hand corner.

Publication bias occurs because favourable results have a higher probability of being published compared with nonsignificant results. The absence of the latter may result in overestimations in meta-analyses. We analysed publication bias in our study using funnel plots, which show the effect measure of each study on the X axis and a measure of precision, such as the standard error, on the Y axis. A meta-analysis without publication bias shows a point cloud in the shape of an inverted funnel. Based on this assumption, we performed a nonparametric analysis called trim-and-fill, estimating the number of missing studies and recalculating the intervention effect after including these new “filled” studies [21]. We also used a less conservative method, proposed by Copas et al. [22], for estimating the number and results of the missing studies.

Meta-regression was used to establish whether the type or duration of interventions affected effect size. The included studies covered six intervention types: Basic education and general counselling on healthy living and diet (Int.1), specific diet/changes in diet and food intake (Int.2), behavioural changes/coaching (Int.3), physical exercise education and/or training (Int.4), stress and/or sleep management (Int.5) and internet/social networks (Int.6).

The authors of the selected articles presented their results in three different ways: mean (± standard deviation) before and after the intervention, difference in means (± standard deviation) before and after the intervention, and difference in medians (with interquartile range) before and after the interventions. For the meta-analysis we used the second method (mean difference ± standard deviation). To do this, we calculated the weighted difference in means and standard deviation for the first case, and for results presented as medians and interquartile ranges, we approximated the mean and standard deviation according to the methods of Luo et al. [23] and Wan et al. [24], respectively.

The statistical analysis was performed using the R packages meta (Version 4.10-0) [25] and metasens (Version 0.4-0) [26].

## 3. Results

### 3.1. Systematic Review

**Figure 1:**
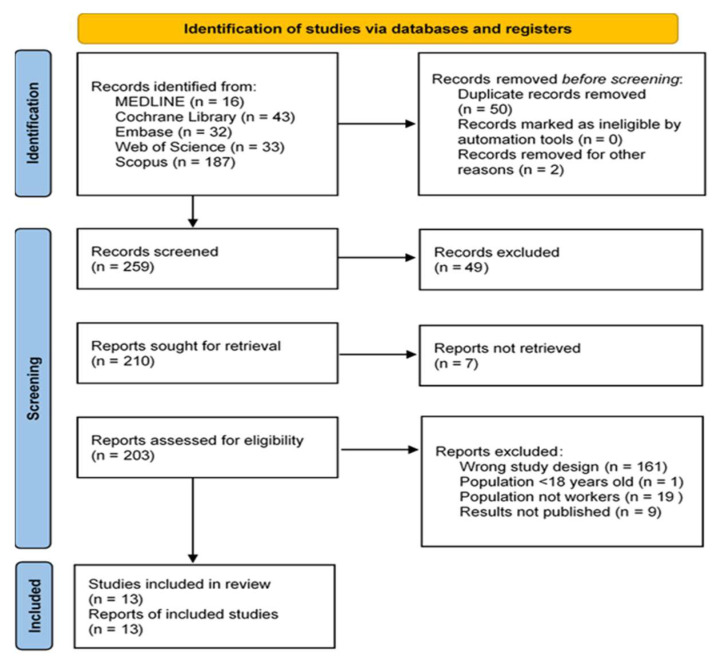
Study Selection Procedure. From: Page MJ, McKenzie JE, Bossuyt PM, Boutron I, Hoffmann TC, Mulrow CD, et al. The PRISMA 2020 statement: an updated guideline for reporting systematic reviews. BMJ. 2021; 372:n71. doi: 10.1136/bmj.n71. For more information, visit: http://www.prisma-statement.org/ (accessed on 14 September 2021).

Applying the study criteria listed above, we recovered 311 records: 16 in MEDLINE, 43 in the Cochrane Library, 32 in Embase, 33 in the Web of Science and 187 in Scopus. (Figure 1).

Of the 311 records identified, 50 were duplicates and 2 were redundant, leaving 259 records for screening. A further 56 records were discarded after screening, leaving 203 for full text review. Of the articles excluded during the screening stage, 16 were not related to MetS, 8 did not measure the relationship between occupational health and MetS, 14 did not include a dietary intervention, 7 had no available full text, and 11 were published in other languages (Chinese, Japanese, Korean) (Figure 1).

In the second stage, 190 records were discarded, because 161 were not clinical trials or comparative studies, 19 did not include a population of workers, and data were not shown in 9. Only one study was carried out in young people.

After removing duplicates, applying the inclusion and exclusion criteria, reviewing the references of the included articles and relevant reviews, and consulting experts, we included 13 documents [27,28,29,30,31,32,33,34,35,36,37,38,39] (Table 1).

When evaluating the quality of the 13 select articles using the CONSORT questionnaire, the scores ranged from 9.5 to 20.5 with a median of 13.5 (Table 2). The half-life according to Burton and Kebler was six years, and the Price Index was 30.8%, which implies that only four articles were less than 5 years old.

The inter-rater agreement in the selection of articles, measured by Cohen’s Kappa coefficient, was 62% (*p* < 0.001).

Regarding study design, all of the articles included in the review were clinical trials, 12 randomised [27,28,29,30,31,32,33,35,36,37,38,39] and one nonrandomised [34]. All manuscripts were written in English, and the studies had been conducted in seven different countries. The USA [30,31,32,36,39] and Japan [34,37,38] were the biggest contributors. With respect to the clinical criteria used to evaluate metabolic syndrome, studies [31,32,36,39] (four of the five studies conducted in the USA) used the Third Report of the National Cholesterol Education Program Adult Treatment Panel (NCEP-ATP-III) [40]. Shrivastava et al. [29] and Inoue et al. [34] used these same criteria. The remaining studies used heterogeneous classifications. The criteria of Woo et al. [27] were based on but not defined by the guidelines for treating adult diseases (National Cholesterol Education Program 2002), published by the USA National Heart, Lung and Blood Institute [40]. Proeschold-Bell et al. [30] used the same classification to categorise BMI, and also referred to the MetS definition of the International Diabetes Federation [41], as did Puhkala et al. [33]. Chen et al. [35] and Nanri et al. [37] employed criteria specific to their countries, Taiwan and Japan, respectively [42,43]. Studies [28,38] did not specify the clinical indicators used to evaluate MetS.

The number of workers included in the different studies varied greatly, from 35 [34] to 1390 [31]. The male to female ratio also varied: three studies [34,37,38] included only men and one [35] included only women. Men predominated in studies [27,28,29], and women in [31,35,36,39]. The male to female ratio was not reported by Woo et al. [27] or by Puhkala et al. [33]. In most of the studies, the average age of participants was around 50 years. 

The 13 included studies covered several places of work, though most were conducted with office or administrative workers. The participants of six studies worked either in a hospital [27,28,32,39] a medical company [31] or insurance company [38]. Shrivastava et al. [29], Inoue et al. [34] and Nanri et al. [37] included public or private sector office workers, without providing further details. Proeschold et al. [35] recruited United Methodist clergymen and Puhkala et al. [33] studied long-distance bus and truck drivers. Allen et al. [36] enrolled university employees and Chen et al. [35] do not specify beyond the term “career women” in their article.

All but two studies included individuals with overweight or obesity, either as an isolated MetS risk factor [28,29,30,32,33] or combined with other risk factors [27,31,35,36,37,38]. Inoue at al [34] included workers who did not perform daily physical exercise and who usually travelled by train or bus. In the inclusion criteria of [34], workers’ state of health was not taken into account.

Follow-up times ranged from three months [27,34,35] to three years [28], with the most common being 12 months [31,32,33,36,39]. 

#### 3.1.1. Dietary Interventions

The included studies applied a variety of interventions led by experts who were lifestyle coaches or health managers [27,30,31,32,35,36], nutritionists or dieticians [27,29,32,37,39], nurses [28,32,37,39], physical trainers [29,38,39] or a physiotherapist [33]. In the study by Inoue and colleagues [34], a cook in the staff cafeteria led the intervention. Table 3 summarises this information and the different components of each intervention. All but one study [34] used a multicomponent strategy, classified into four main groups: 1. Basic education and general counselling on healthy habits and diet; 2. Specific diet or changes in diet and food intake; 3. Motivational changes and/or coaching; 4. Physical exercise and stress and/or sleep management. Most interventions were fully or partially implemented through online platforms and/or social networks. 

#### 3.1.2. Basic Education and General Counselling on Healthy Habits and Diet

Six of the 13 included studies implemented an intervention focused on offering participants basic education and general counselling on healthy habits and diet [27,29,30,33,36,37]. In general, these interventions consisted of educative sessions aimed at improving participants’ knowledge of the importance of leading a healthy lifestyle. The main nutrition topics included in the sessions were related to portion control; a healthy plate model; balancing fats, proteins and carbohydrates; increasing intake of fruits and vegetables; food types in terms of glycaemic index; reducing sugar or alcohol consumption; and sampling of healthy foods.

Woo et al. [27] and Allen et al. [36] also included sessions focused on explaining MetS risk factors or how to reduce risk of cardiovascular disease, diabetes or hypertension.

#### 3.1.3. Specific Diet/Changes in Diet and Food Intake

Five studies included specific diets or dietary and/or nutritional changes, implemented through different interventions. Maruyama et al. [38] and Chen et al. [35] proposed a personalised nutritional plan based on previous analysis of participants’ dietary and nutritional habits, setting specific goals and recording calorie intake.

Inoue et al. [34], meanwhile, evaluated the impact of a healthy Japanese diet on the prevention and reduction of metabolic syndrome. Puhkala et al. [33] used the healthy eating plate model developed in 2011 by Harvard University [44] to help change participants’ dietary habits. Racette et al. [39] used the Weight Watchers© model, consisting of group sessions, incentives and a healthy snack cart.

#### 3.1.4. Behavioural Changes or Motivational Coaching

Some interventions were based on behavioural change models that aim to improve workers’ health behaviours through motivation, changing health beliefs and empowering self-efficacy. Woo et al. [27] used a programme based on Rosentock’s 1990 health belief model [45], while Racette et al. [34] used the transtheoretical model of behaviour change developed by Prochaska and DiClemente in 1983 [46]. Proeschold-Bell et al. [30] delivered a stress management programme developed by Williams LifeSkills [47] to improve interpersonal relationship skills, and also offered theological content supporting healthy behaviour. Kramer et al. [32] and Nanri et al. [37] also used programmes based on behavioural change theory to modify participants’ lifestyles.

In three studies [27,31,38], coaching techniques were included in the interventions to improve participants’ commitment and help them to maintain behavioural changes [48].

#### 3.1.5. Other Interventions: Physical Activity, Stress Management and Sleep Hygiene

Eleven of the 13 interventions included a component related to physical exercise. Some were centred on regular physical counselling and increasing daily steps [27,28,35,36,37], while others included physical activity training sessions or personalized exercise plans [29,31,38]. Kempf et al. [28] included telemonitoring of participants’ physical activity

#### 3.1.6. Use of Internet and Social Networks

Although this aspect is not a type of intervention per se, the interventions in most studies were fully or partially implemented through web resources, platforms or social networks to facilitate follow-up and communication with participants. These digital tools included online health platforms and study-specific web portals [23,24,26,30,31,33,35], as well as apps or e-mail reminders [26,28]. Shrivastava et al. [29] tracked adherence to lifestyle changes through smartphone messages, a digital health platform, e-mail and phone calls. Regarding the use of social networks, Woo et al. [27] used an instant messaging app called KakaoTalk, and Steinberg et al. [31] used Skype. Most studies combined telematic resources (digital platforms, mobile applications, specific websites) with face-to-face activities in participants’ workplaces [29,30,32,33,38,39].

Three studies used telematic means only [27,28,36], while the interventions of Inoue et al. [34] and Nanri et al. [37] included only the traditional face-to-face method. 

#### 3.1.7. Results of Dietary Interventions

The effect of the dietary interventions was measured through changes in the different variables proposed as health markers: WC, BMI, TC, HDL-c, LDL-c, TG, SBP, DBP and FBG. Table 1 summarises these results.

1. Improvement in Anthropometric Measurements

Waist Circumference (WC, cm)

All but two studies [30,39] included this parameter. The mean pre-intervention values recorded in [27,29,32,33,34,35,36,37] ranged from 85.7 ± 7.1 cm [34] to 114.9 ± 10.3 cm [33] and post-intervention values ranged from 84.8 ± 7.4 cm [34] to 109.1 ± 3.7 cm [33]. 

Seven of the studies found significant reductions [27,29,31,32,35,37,38].

Body Mass Index (BMI, kg/m^2^)

In all but two studies [31,35], researchers reported participants’ BMI values as an outcome measure. Eight studies used a before-and-after design [23,27,28,36,39] recording BMI at the beginning and end of the intervention. Mean initial BMI values were between 24 ± 2.4 kg/m^2^ [34] and 34.5 ± 9.7 kg/m^2^ [39], while post-intervention values ranged from 23.8 ± 2.5 kg/m^2^ [34] to 34.1 ± 9.8 kg/m^2^ [39].

Only one study reported healthy pre-intervention BMI values (according to WHO guidelines; ≤25 kg/m^2^) in one of its intervention arms [34]. Five studies reported a mean BMI value within the overweight range (≥25 kg/m^2^) [27,29,36,37,38]. Another five studies reported a mean BMI value within the obese range (≥30 kg/m^2^) [28,30,32,33,39]. In six studies, researchers observed significant reductions [27,28,29,32,37,38].

2. Improvements in Lipid Profile

Total Cholesterol (TC, mg/dL)

In all but two studies [30,35], authors recorded total cholesterol as an outcome measure. Among the studies that reported mean total cholesterol [27,29,32,34,35,36,37,38,39], this value ranged from 192.74 ± 30.54 mg/dL [27] to 229.6 ± 26.9 mg/dL [37] before the interventions and from 175.00 ± 34.38 mg/dL [27] to 217 ± 31.1 mg/dL [37] after. 

Seven of the studies found significant reductions [27,29,31,34,37,38,39].

High-Density Lipoprotein Cholesterol (HDL-c, mg/dL)

All studies included HDL levels as an outcome measure, with the exception of Steinberg et al. [31]. Nine studies collected data on pre and post-intervention HDL-c [27,29,32,33,34,35,36,37,39], with mean baseline values ranging from 40.1 ± 8.32 mg/dL [29] to 59 ± 11 mg/dL [34] and mean post-intervention values ranging from 40.49 ± 8.1 mg/dL [24] to 62 ± 18 mg/dL [39]. Baseline HDL-c was ≥ 40 mg/dL in all studies that applied NCEP-ATP-III criteria [40].

Low-Density Lipoprotein Cholesterol (LDL-c, mg/dL)

Ten studies reported LDL-c as an outcome measure [27,28,29,32,33,34,36,37,38,39]. Seven studies provided pre and post LDL-c levels [27,29,32,34,36,38,39], with mean values ranging from 114.3 ± 31.3 mg/dL [32] to 136 ± 30 mg/dL [34] before the intervention, and 106 ± 26 mg/dL [39] to 132 ± 26 mg/dL [34] after. Most baseline mean values were at a suboptimal level, as defined by NCEP-ATP-III [28,40].

Three studies reported significant reductions for this parameter [27,38,39].

Triglycerides (TG, mg/dL)

All studies included triglyceride levels as an outcome measure, and eight provided pre and post intervention data [27,29,34,35,36,37,38,39]. Mean baseline values were between 115 ± 59 mg/dL [39] and 208 ± 116 mg/dL [34], and final values ranged from 106 ± 13.6 mg/dL [38] to 181 ± 67 mg/dL [34]. 

According to the NCEP-ATP-III classification [40], 50% of the studies showed TG levels indicative of MetS (>150 mg/dL). 

Significant reductions were observed in six studies [27,29,31,32,37,38].

3. Changes in Blood Pressure Readings

Of the 13 included studies, 11 reported blood pressure as an outcome measure [27,28,29,32,33,34,35,36,37,38,39]. Ten studies adopted a before-and-after design, recording baseline and final values [27,28,29,32,34,35,36,37,38,39]. Mean systolic blood pressure before the intervention ranged from 120.07 ± 12 mm Hg [27] to 146 ± 14.4 mm Hg [28] and after the intervention from 118.80 ± 4.7 mm Hg [32] to 138.1 ± 14.9 mm Hg [34]. Mean baseline diastolic blood pressure values were between 76.5 ± 12.5 mm Hg [30] and 90.5 ± 11.9 mm Hg [34], and final values ranged from 73.5 ± 10.1 mm Hg [35] to 91.2 ± 11.4 mm Hg [37]. 

Three articles recorded significant reductions in SBP [28,34,39] and four recorded significant reductions in DBP [28,32,38,39].

4. Changes in Fasting Blood Glucose (FBG, mg/dL)

Fasting blood glucose was measured to assess diabetes risk. Samples were taken between 7:00 and 8:00 in the morning. Most data were provided in mg/dL and the rest we transformed from mmol/L to mg/dL.

Of the included articles, 10 collected data for this parameter [27,29,32,33,34,35,36,37,38,39]. FBG levels were measured before and after the interventions, with mean initial values between 90.1 ± 7.6 mm/dL [37] and 118 ± 28 mm/dL [34], and final values between 90.6 ± 7.7 mg/dL [31] and 111 ± 18 mg/dL [29]. 

Three studies reported significant reductions in FBG [30,33,34].

5. Improvements in Prevalence of MetS or in Number of Risk Factors for MetS

Four studies analysed the change in MetS prevalence after the intervention [30,33,37,39]. In the study by Proeschold et al. [30], for participants with at least one follow-up measurement, reductions in MetS prevalence were observed in all cohorts at 24 months, ranging from 3.7 to 6.6 percentage points. In Puhkala et al. [33], the between-group differences in the prevalence curves covering 24 months of intervention (evaluation at 0, 12 and 24 months) were not statistically significant (*p* = 0.11). In Nanri et al. [37], MetS prevalence decreased for both the intervention and control group, from 100% at the beginning to 65.3% and 62.3%, respectively, at six months. However, the authors found no statistically significant difference when comparing the two groups (*p* = 0.75). Racette et al. [39] found reductions in MetS prevalence in both groups, from 38% to 25% in the intervention group and from 29% to 18% in the control group. For participants who received the intervention, the authors attributed this improvement to a reduction in blood pressure and increase in HDL-c.

Some studies assessed the change in a cluster of risk factors for MetS [29,35,36]. Most studies stated which definition they had used for metabolic syndrome. 

Shrivastava et al. [29] found that in their intervention group, the number of people with three, four or five risk factors reduced, while the number of people with one or two risk factors increased. Few participants in either group reduced their risk factors to zero. Specifically, the number of subjects with three risk factors lowered from 27% to 19% in the intervention group compared to an increase from 21% to 22% in the control group. For four risk factors, this change was 14% to 8% in the intervention group versus 18% to 15% in the control group, and for five risk factors, 7% to 3% versus 4% to 5%. In Chen et al. [35] the intervention group showed better results in terms of mean number of MetS components compared with the control group (−0.6 vs. 0.1, *p* < 0.05).

6. Health Beliefs, Health Promotion Behaviours and Self-Efficacy

The programme applied by Woo et al. [27] was based on Rosentock’s 1990 health belief model [45], and health promotion behaviours of workers in the intervention group showed greater improvement compared with the control group. In Racette et al. [39], all components in the intervention were based on the transtheoretical model of behaviour change (Prochaska and DiClemente, 1983) [46], which centres on individuals’ tendency to adopt a healthy or unhealthy behaviours. Many participants made behavioural changes such as increasing physical activity and improving eating habits, which contributed to clinically significant health improvements. The intervention programme with coaching adopted by Maruyama et al. [38] obtained improvements in dietary habits as well as in 14 of 17 clinical parameters.

7. Changes in Food Group or Diet/Nutrition Intake

Shrivastava et al. [29] found a significant change in dietary behaviour after the intervention, with participants choosing healthier options. Consequently, they observed a decrease in total daily caloric intake (from 1823 ± 353 to 1665 ± 367 kcal) and proportion of fat in total energy (from 35% to 32%).

In the study by Inoue et al. [34], the intervention group who consumed fewer than 50 of a potential 61 healthy Japanese-style lunches had reduced their total daily intake of energy and of carbohydrates at three months compared with baseline (energy: 2554 ± 392 kcal vs. 2104 ± 393 kcal, *p* = 0.042; carbohydrate: 359.6 ± 85.2 g versus 295.8 ± 45.3 g). Fibre intake increased significantly in the group who had 51 or more Japanese lunches (total dietary fibre: 15.3 ± 5.2 g vs. 30.4 ± 20.9 g, *p* = 0.047; total vegetables: 292.4 ± 146.6 g vs. 411.1 ± 155.9 g, *p* = 0.035).

After implementing a programme designed to promote healthy dietary habits and physical activity, Maruyama et al. [38] observed changes in typically consumed foods in the intervention group (*p* > 0.01). The magnitude of the intervention effect was 0.31 for food group A (fish, soya bean/soya products, green/deep-yellow vegetables, white vegetables and mushrooms/seaweed/konnyaku) and 0.35 for food group B (large portions of grains such as rice/bread/noodles, confectionery, sweet drinks, fatty meats, meat products, butter/margarine/dressing/mayonnaise, eggs/liver, fried foods, pickles, soup and alcoholic drinks).

8. Changes in Total Daily Physical Activity

Eight studies included results related to physical activity [29,32,33,35,36,37,38,39]. Two studies measured post-intervention physical activity in terms of steps [33,38] and three others in terms of minutes of exercise per week [32,37,39]. 

Chen et al. [35] performed a group and time interaction analysis at 1.5 months, finding better exercise scores in the intervention group than in the control group. Allen et al. [36] suggested reaching a goal of 10,000 daily steps to maintain good health. The intervention group had achieved a 31% improvement after 12 months compared with baseline. 

### 3.2. Meta-Analysis

In the meta-analysis we included 11 studies and 22 study groups who received interventions.

#### 3.2.1. Effect Size

**Figure 2:**
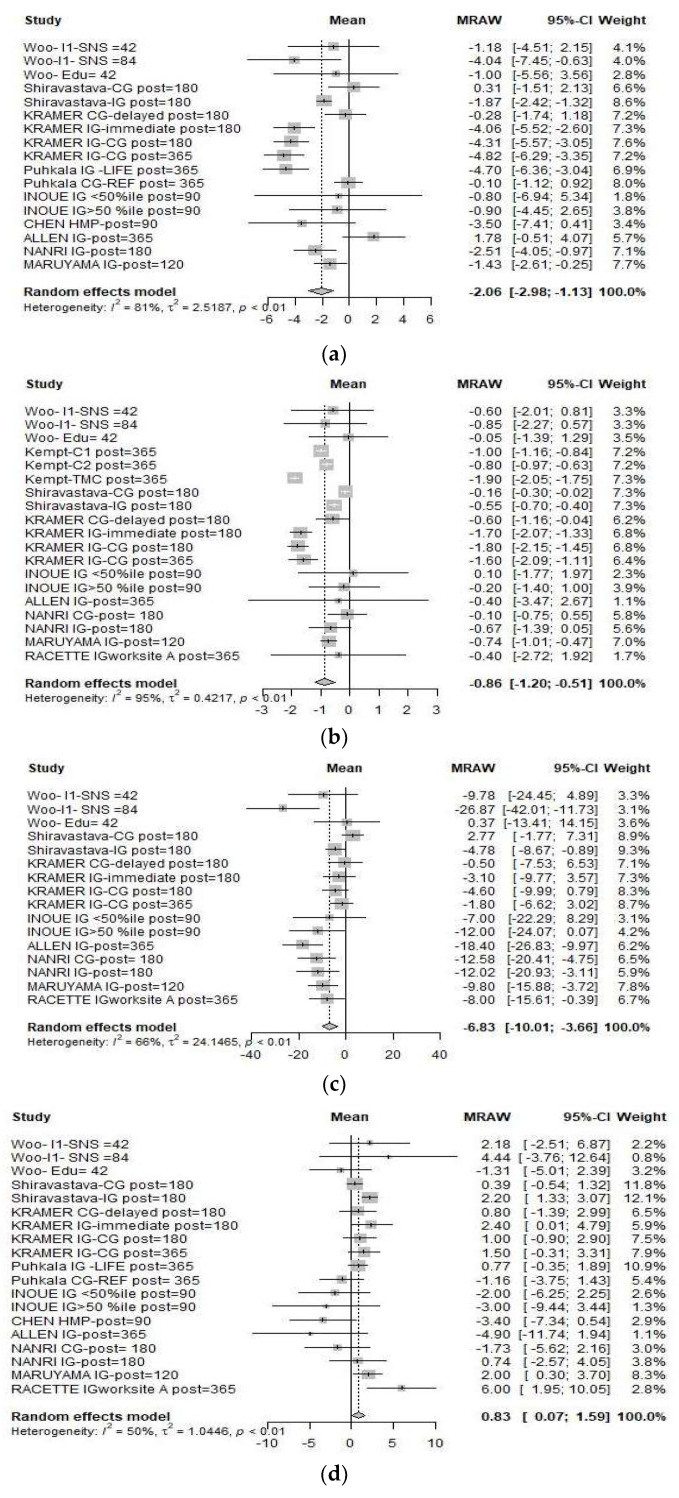

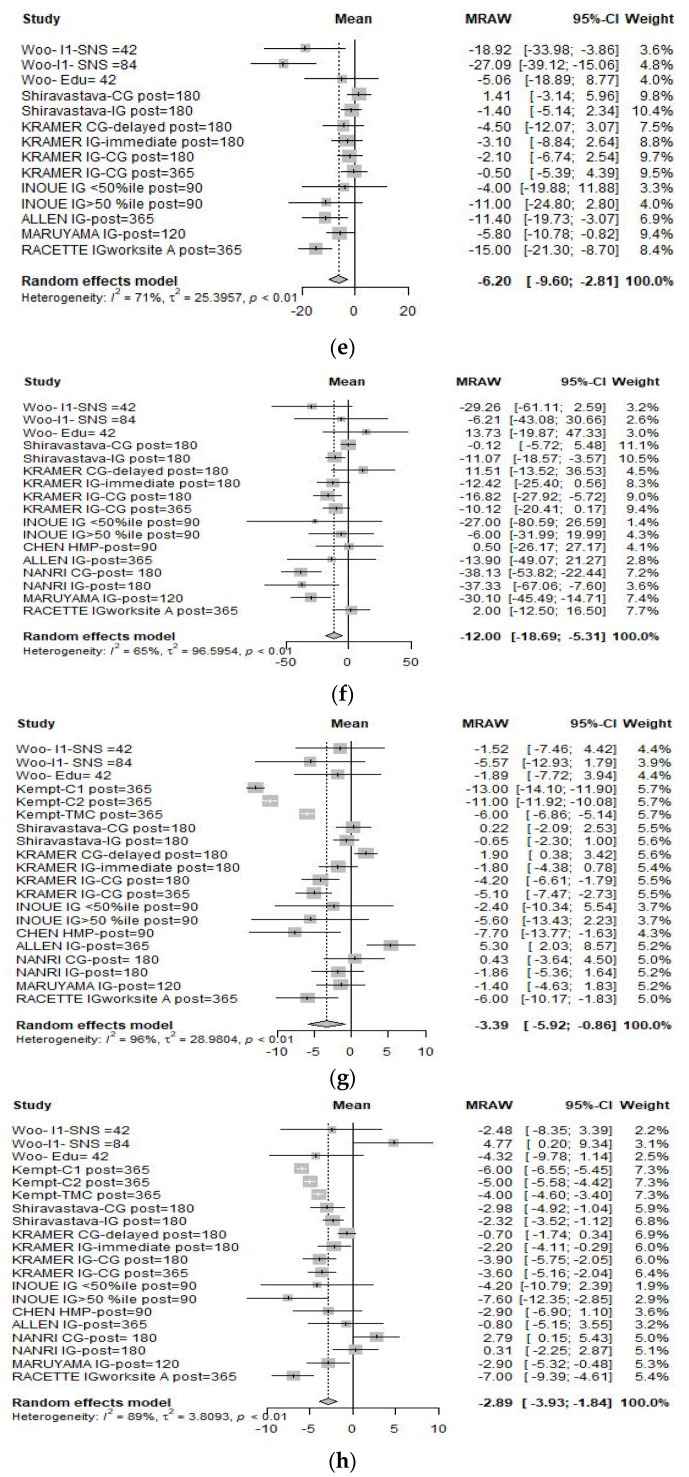

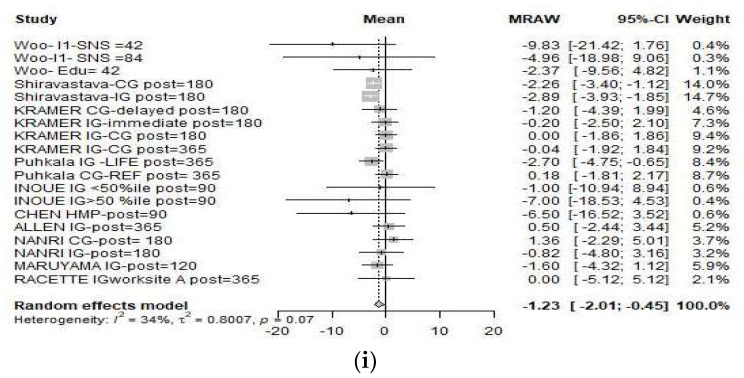
Forest plots for (**a**) waist circumference, (**b**) body mass index (**c**) total cholesterol, (**d**) high-density lipoprotein cholesterol, (**e**) low-density lipoprotein cholesterol, (**f**) triglycerides, (**g**) systolic blood pressure, (**h**) diastolic blood pressure, (**i**) fasting blood glucose.

The effect sizes calculated in the meta-analysis are presented in Figure 2, together with the heterogeneity test results.

#### 3.2.2. Heterogeneity of the Included Studies

The analysis of the nine parameters included showed that overall, the interventions have changed the baseline values, achieving reductions in all except HDL, which increased significantly (Figure 2d). It should be noted, however, that eight of the nine variables showed considerable heterogeneity. We therefore considered it appropriate to examine this heterogeneity between studies. Table 4 presents the results of the leave-one-out analysis.

**Figure 3:**
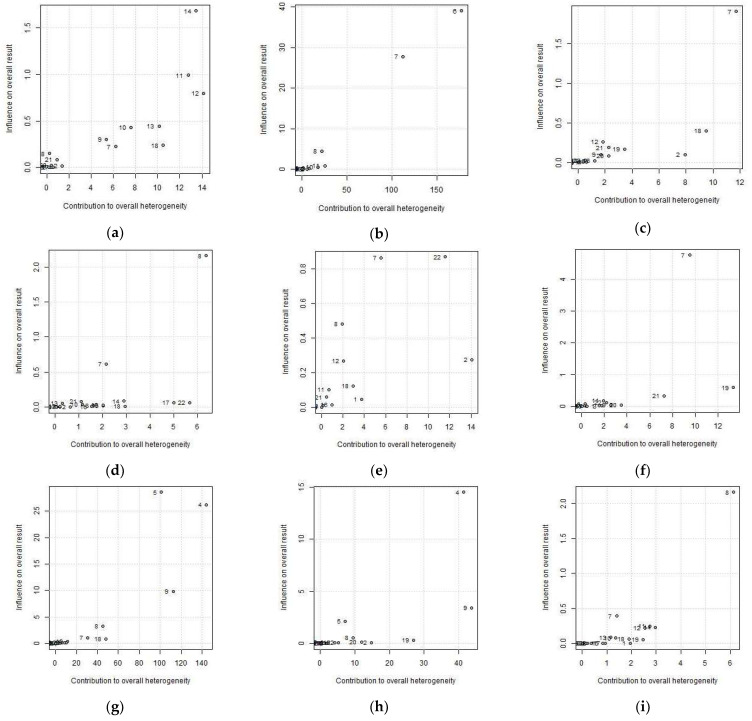
Baujat plots for (**a**) waist circumference (WC), (**b**) body mass index (BMI), (**c**) total cholesterol (TC), (**d**) high-density lipoprotein cholesterol (HDL-c), (**e**) low-density lipoprotein cholesterol (LDL-c), (**f**) triglycerides (TG), (**g**) systolic blood pressure (SBP), (**h**) diastolic blood pressure (DBP), (**i**) fasting blood glucose (FBG). The number correspond to the items in the ID column of Table 4.

The study groups with the greatest heterogeneity contributions are the two study groups of Shrivastava et al. [29] and one group from the Kempf et al. study [28], specifically control group 1 at 365 days. Omitting the Shrivastava et al. groups produces the greatest reduction in heterogeneity in the variables TC, TG, HDL-c and FBG, while the variables most affected by the Kempf et al. control group are SBP and DBP. However, the change in both SBP and DBP does not exceed 5% when this study is omitted (1.4% and 4.3%, respectively). Similarly, the leave-one-out analysis reveals subtle changes in the heterogeneity of the variables WC and BMI. Regarding LDL-c, one of the results collected by Woo et al. [27] at 84 days produces a change in heterogeneity exceeding 10%. However, these changes in heterogeneity should be accompanied by the influence on the final result. Figure 3 depicts Baujat plots, which plot the heterogeneity contribution of each study group against its influence on the pooled result. The scales are relative, meaning the graph only serves to identify studies with considerable influence on both heterogeneity and on the final result. The numbers shown in the figure correspond to the items in the ID column of Table 4.

In Figure 3 we can see that for the four variables that may compromise heterogeneity (TC, HDL, LDL and TG), only the Shrivastava et al. study groups occupy the upper right-hand corner, reflecting the greatest contribution to heterogeneity and influence on the effect.

#### 3.2.3. Heterogeneity Due to Missing Studies (Publication Bias)

**Figure 4:**
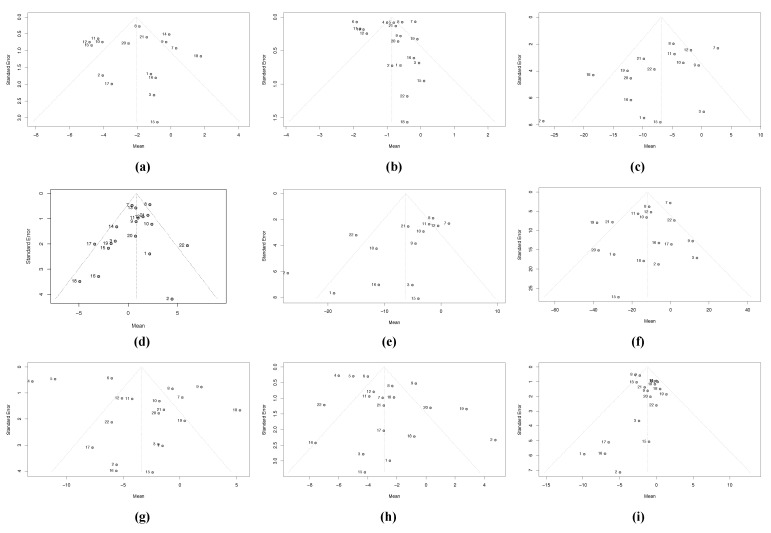
Funnel plots for (**a**) waist circumference (WC), (**b**) body mass index (BMI), (**c**) total cholesterol (TC), (**d**) high-density lipoprotein cholesterol (HDL-c), (**e**) low-density lipoprotein cholesterol (LDL-c), (**f**) triglycerides (TG), (**g**) systolic blood pressure (SBP), (**h**) diastolic blood pressure (DBP), (**i**) fasting blood glucose (FBG).

Another possible source of heterogeneity is publication bias. For this reason, we created funnel plots and analysed their symmetry. These graphs are shown in Figure 4.

Table 5 shows the results of the most classic methods for estimating the number of missing studies and the influence they could have on the final result.

The trim-and-fill method shows publication bias in seven of the nine variables. All seven effect sizes are reduced, but only those of HDL and TG are nonsignificant after this adjustment. With the Copas method, only TC and LDL show publication bias, and the adjusted effect sizes, while reduced, remain statistically significant (Table 5).

#### 3.2.4. Moderator Analysis or Meta-Regression

Heterogeneity may also be influenced by covariates or moderators. Table 6 shows the influence of the six intervention types and intervention duration.

The duration of treatment is associated with a reduction of DBP and an increase in FBG. The interventions with the greatest impact on effects are types 3 and 4, which reduce BMI and increase HDL. Intervention 3 also increases FBG. Type 1 interventions increase BMI and blood pressure (SBP and DBP).

## 4. Discussion

To the best of our knowledge, this is the first systematic review and meta-analysis to group and synthesise the available scientific literature and analyse the characteristics and effects of dietary interventions aimed at reducing MetS risk in the working population. The interventions with the greatest effect are those that include physical activity and that focus on health beliefs and behaviours and workers’ motivations. Although many clinical results reflect only a modest impact on MetS risk, our results suggest that interventions are beneficial on the whole.

We restricted our search to clinical trials and comparative studies because we aimed to establish a cause–effect relationship [49]. In the end we included 13 studies. The low obsolescence of these studies reflects their validity and the timeliness of our review; the data obtained (Price Index and Burton–Kebler index) indicate lower obsolescence than typically found in nutrition science bibliometric results, showing that workplace dietary interventions for reducing MetS risk is an emerging topic of interest.

Although we found substantial methodological and clinical heterogeneity between the 13 studies, we were able to include 11 in the meta-analysis. Because the sample sizes were generally small (*n* < 125), this meta-analysis was needed to obtain more robust conclusions.

The study participants were aged between 30 and 60 years, as would be expected in a working population. Our review included several different work places, but most participants were office workers or had jobs that did not require great physical effort (long-distance drivers, clergy, health workers), and most had overweight, obesity and/or one or more other MetS risk factors. These workers perform sedentary activities, and this together with inadequate diet can lead to overweight and obesity, increasing MetS prevalence [10]. In addition, the high responsibility of these jobs and the complex tasks often involved can generate considerable pressure and lead to occupational stress, another risk factor for metabolic syndrome [50].

In the studies included in our review, the choice of MetS definition depended on the country where the study took place. In the absence of a single definition, several closely related but individual definitions have been proposed for MetS. Four studies used the criteria of the National Cholesterol Education Program Adult Treatment Panel III (NCEP-ATP-III) [40]. These criteria are straightforward and readily measurable, making them easy to apply clinically and epidemiologically [51]. For this reason, the NCEP-ATP-III definition is among the most widely used for MetS [51]. Not all studies stated which definition they applied; some simply evaluated the presence of risk factors such as insulin resistance, obesity, atherogenic dyslipidaemia and hypertension. In any case, all studies largely conformed to the harmonised classification proposed by Alberti et al. in 2009 [2]. The criteria listed in this classification constituted the main tools for measuring the effect of interventions, in some cases together with questionnaires on food intake, physical activity or stress.

Most follow up periods lasted one year or less and were not established according to any standard. Duration was a major limitation of many studies. Several authors recognised that longer follow-up times were needed to determine the sustainability of lifestyle changes and to be able to correct deviations accordingly. [29,37]. One strength was that most interventions were led by health professionals (doctors, nurses, dieticians), which added scientific rigour to the programmes.

The dietary interventions were grouped into six main types: 1. Basic education and general counselling on healthy habits and diet; 2. Specific diet/changes in diet and food intake; 3. Behavioural change and coaching; 4. Physical exercise education and training; 5. Stress management. 6. Internet and social networks.

All but one of the programmes included more than one intervention type. This made it difficult to isolate the effect of each type on the final result, justifying the meta-regression we carried out.

Our results show that most intervention groups achieved a significant improvement in the parameters assessed. Considered together, the interventions were beneficial. However, the clinical impact of these improvements on workers’ health was moderate. For example, each 1 mg/dL increase in HDL-c is thought to reduce risk of coronary death by 6%, regardless of LDL-c values [52]. Regarding blood pressure, a recent meta-analysis including data from 48 clinical trials showed that a 5 mm Hg reduction in systolic blood pressure reduces the risk of major cardiovascular events by around 10%, regardless of cardiovascular disease history, and even at normal or high-normal blood pressure levels [53]. With respect to waist circumference (another diagnostic criteria of MetS), previous studies carried out in women show that a 5 cm reduction is related to at least 10% improvement in at least one cardiovascular risk factor [54]. When we consider all this evidence, we see that the differences recorded in the reviewed studies scarcely improve workers’ risk profile. It therefore seems necessary to establish which interventions are most effective for reducing MetS risk and to analyse these interventions in depth with the aim of improving future clinical results.

In our review, the interventions with the greatest impact on effect size were those that used coaching techniques and/or that applied behavioural change theory to modify eating and lifestyle habits, and those that promoted physical exercise. This result reinforces the evidence that the most effective approach to MetS is achieved by targeting diet and physical activity [55], and suggests that to optimise the results of MetS-focused workplace programmes, dietary interventions should be combined with physical exercise. This finding was to be expected: physical exercise is a powerful tool in the fight to prevent and treat numerous chronic diseases [56]. Physical activity impacts on the components of MetS, such as cardiovascular risk, blood pressure, lipid profile and blood glucose levels. Previous studies have shown a link between physical exercise and improved MetS. Haufe et al. [57] conducted a randomized controlled trial in workers at a motor vehicle company, finding that a programme of regular and telemonitored physical activity reduced metabolic syndrome and improved autonomy in the workers who took part. Tsai et al. [58] assessed the effect of a 12-week physical exercise programme on MetS components in bank and insurance workers. Their results show that intense exercise helps to improve blood pressure levels and waist circumference.

In contrast, the most common interventions, which were generally group-based and focused on offering information to participants on healthy habits and lifestyle, led to an increase in BMI and blood pressure (both systolic and diastolic). This finding has been described by other authors, who state that subjects often seem to make wrong decisions when they receive information on health risk factors [53]. Interventions must go further than simply providing information and focus more on the concept of literacy, empowering participants and offering them tools to process the information relevant to their health.

Low health literary is associated with a lack of understanding of concepts, worse management of disease and self-care activities, lower use of preventive measures, errors in compliance with treatment, and difficulty understanding health advice [59]. Giving patients information is not enough; they need to understand the information, be able to identify information that is appropriate and true, then be able to interpret and apply it according to their circumstances and personal needs [60]. To ensure educational interventions are successful, or at least to prevent a contrary effect, planners should consider the varying levels of literacy that may exist among the workers of a single company.

Our results highlight the importance of motivating and empowering workers who receive a behavioural change intervention, as well as addressing their health beliefs. The interventions that used coaching techniques or that centred on behavioural change theory had the greatest impact on the effects. These cognitive-behavioural programmes aim to change beliefs and motivate workers to acquire and maintain preventive practices. They are personalised and take into account any obstacles to the application and maintenance of preventive practices, setting specific goals for each worker, and typically following up on their achievement [48]. Several studies vouch for the effectiveness of coaching in the clinical setting. A randomised controlled trial demonstrated that this approach, compared with traditional care, significantly improved HbA1c levels in patients with diabetes [61]. A recent meta-analysis of randomised controlled trials found that behavioural treatment strategies improved adherence to lifestyle intervention programmes in adults with obesity [62]. The authors concluded that these strategies should be routinely incorporated into lifestyle interventions and obesity management and weight loss programmes to improve commitment and adherence.

Among the studies included in our review, Shrivastava et al. [29] incorporated several successful components, which probably explains why this study contributed the most to heterogeneity in certain parameters, such as TC, TG, HDL and FBG. 1. The interventions were intensive, with considerable follow-up, and were led by a team of doctors, nutritionists and personal trainers. 2. They focused on raising awareness among workers and improving knowledge, attitudes and practices to achieve the desired results. 3. The programme included sessions every two weeks on healthy habits, diet and physical activity. These sessions covered eating out, cooking methods, reading food labels and meals during festive seasons. 4. The participants received personalised advice and reinforcement. 5. Practical physical activity sessions were provided and participants were encouraged to maintain their new habits. 6. Participants were offered occupational stress management sessions, as stress is another risk factor for MetS [63]. Adherence to lifestyle changes was monitored through individual interviews and digital tools such as smartphones, an online platform, emails and repeated phone calls. This type of follow-up is missing from all the other studies. The authors achieved not only a considerable reduction in the parameters of interest, but also a change in clustering of MetS profile status, which is the ultimate objective of this type of intervention. Theirs could serve as a reference for future worksite health promotion programmes.

Previous systematic reviews have assessed the effect of lifestyle interventions on MetS [64,65] in the general population with and without MetS. The results of these reviews indicate that lifestyle modification helps to reduce MetS prevalence and the severity of its individual components. For example, Van Namen et al. [5]. carried out a systematic review and meta-regression of 15 papers reporting data on 1160 participants from 10 randomised controlled trials, to investigate the effects of lifestyle interventions—including both dietary changes and supervised exercise on outcomes for people with MetS. Compared with usual care, lifestyle interventions achieved significant improvements in waist circumference (−4.9 cm, 95% CI −8.0 to −1.7), systolic blood pressure (−6.5 mmHg, 95% CI −10.7 to −2.3), diastolic blood pressure (−1.9 mmHg, 95% CI −3.6 to −0.2), triglycerides (SMD −0.46, 95% CI −0.88 to −0.04) and fasting glucose (SMD −0.68, 95% CI −1.20 to −0.15). The authors conclude that health services should consider implementing lifestyle intervention programs for people with metabolic syndrome to improve health outcomes and prevent progression to chronic disease.

In view of these findings, both health services and workplaces appear to constitute ideal settings for implementing lifestyle intervention programmes to improve health outcomes and prevent progression to chronic diseases. This conclusion is supported by international organisations such as the WHO, which has developed the Healthy Workplace Model and Framework [66], and proposes workplace programmes as one of the key strategies to improve population health. Workplaces have the infrastructure to provide workers with chronic disease prevention interventions at different levels [67]. Interventions in this setting could therefore make a significant contribution towards reducing chronic disease risk at the population level.

## 5. Limitations of the Review

Out study is not without its limitations. One important weakness is that we were unable to retrieve the full text of some articles because they were not published in the journal’s website or did not feature in the main collections. Nor were we able to retrieve these texts through the university library network or by contacting the authors. Another limitation is that the articles did not report on intervention dose or cost-effectiveness of the interventions. This information might be useful for the development of an adequate evidence base to support practice.

In addition, the methodological differences between interventions and the varying profiles of the workers studied made it difficult to interpret the results. Nevertheless, the meta-analysis and meta-regression helped us to explain possible sources of heterogeneity and to analyse and synthesis the information obtained from the different studies.

Lastly, we found some publication bias, though not enough to cancel the effects.

## 6. Implications for Future Research

It is clear that workplaces are important settings for health promotion and disease prevention. In view of our results, it seems necessary to continue investigating which interventions are most effective for preventing MetS and to continue exploring new strategies. Almost all the interventions analysed in this review are individualistic, aimed at raising awareness among workers and educating them on healthy habits. Only Inoue et al. [34] applied what can be considered a mass catering intervention, in which the intervention groups received healthy Japanese-style lunches for three months in the staff cafeteria. Despite the short intervention time, the authors observed a reduction in the parameters assessed, and this reduction was more pronounced in the participants who consumed more healthy lunches. We believe this type of intervention should be studied in greater depth. Regardless of workers’ awareness and motivation, it is often difficult for them to make healthy food choices at work because the food on offer in staff canteens lacks nutritional quality and/or variety. The WHO global strategy on diet, physical activity and health suggests that workplaces facilitate healthy food choices in order to reduce daily risk exposure [68]. The European FOOD programme (Fighting Obesity through Offer and Demand) is an initiative of the European Commission that aims to improve the nutritional quality of foods consumed during working hours as a complementary strategy to individual awareness and education on healthy habits [69]. It proposes that companies, workers and restaurants work together to ensure balanced nutrition at work. This is undoubtedly an interesting avenue to explore in future research.

## 7. Conclusions

It is necessary to improve the efficiency of the dietary interventions aimed at lowering MetS risk in workers. The totality of available evidence suggests work-based interventions have a positive yet modest effect on MetS risk. The interventions with the greatest effect are those that include physical activity and that focus on health beliefs and behaviours and on workers’ motivations. Purely informative or educative interventions are common but have a contrary effect. Employers must take this into account. Our results may help to guide future health promotion programmes aimed at improving workers’ health to reduce risks and possible productivity losses.

## Figures and Tables

**Table 1:** Summary of Included Studies on Dietary Interventions in the Workplace.

Author, Year	Country, MetS Clinical Criteria	Population Studied (N, M to F Ratio, Age, Occupation)	FUP	Health Status Inclusion Criteria	Intervention	Outcome Variables	Results	Conclusions
Woo et al., 2020 [27]	South Korea, NI	N: 68 (IG1 23; IG2 19; CG 26) M/F: NI Age range: 25–60 years Occupation: hospital workers	12 weeks	2 risk factors for MetS or 1 risk factor for CVD	Social Network Service-Based Lifestyle-Modification Programme. IG1: education/counselling about regular physical activity; doctor consultation; nutritional goals. IG2: only educational information. CG: no intervention.	CVD risk factors: BP, WC, BMI, TG, TC, HDL-c and LDL-c. Health beliefs, health promotion behaviours and self-efficacy. Data collected at baseline, 6 weeks and 12 weeks in all 3 groups.	Week 6: IG1 showed significant decrease in WC, BMI, TC, LDL-c, health promotion behaviours and self-efficacy, but not significantly greater than in IG2 or CG. Week 12: IG1 showed significant decrease in WC, BMI, TC, LDL-c. Self-efficacy and health promotion behaviours improved to a greater degree in IG1 than in IG2 and CG.	Programme improved self-efficacy and health behaviour, improving CVD risk factors
Kempf et al., 2019 [28]	Germany, NI	N: 104 (IG 34; CG1 34; CG2 36) M/F: 84/16 Mean age: 50 years Occupation: employees of medical company Boehringer Ingelheim	36 months	Overweight (BMI ≥ 25 kg/m^2^ and/or WC > 94 cm for men or >80 cm for women).	Telemedical coaching focused on controlled weight loss with or without telemonitoring. IG: Telemedical coaching + telemonitoring (scales and pedometer) + weekly then monthly care calls over 12 months. CG1: scales and pedometer from baseline CG2: short coaching phase (monthsmonths 6–9) + scales and pedometer from mth 6.	1. Weight loss after 12 months in all 3 groups 2. Difference in weight loss after 6 months (IG vs. C1 and IG vs. C2). 3. BMI, WC, SBP/DBP, TG, TC, HDL-c, LDL-c, HbA1C, FBG, CRP, eating behaviour, PA. Data collected at 3, 6, 9, 12 and 36 months.	Significant reduction in BMI, SBP/DBP and eating behaviour in all groups. Weight loss after 12 months: IG vs. CG1: −3.6 kg (−7.4; −0.1) (*p* = 0.047); IG vs. C2: −4.2 kg (−7.9; −0.5) (*p* = 0.026) in per-protocol analysis. ≥5%weight loss at 12 months: IG: 63% (*p* = 0.037); CG1: 33%; CG2: 31% in per-protocol analysis. At 36 months: weight loss at 12 months was maintained until 36 months. All 3 groups significantly reduced weight (IG: −8.4 [95% CI −11.3; −5.4] kg; CG1: −4.0 [95% CI −6.6; −1.8] kg; CG2: −3.3 [95% CI −5.8; −0.8] kg). No difference between groups in per-protocol or intention to treat analyses after 3 years	Improvements in SBP, anthropometric measurements and eating behaviour indicate that telemedical coaching with telemonitoring can help to prevent weight gain and improve health.
Shrivastava et al., 2017 [29]	India, NCEP-ATP-III [35]	N: 598 M/F: 87.9%/12.1% Mean age: IG 35.8 years (SD 7.6); CG 39.0 yearsyears (SD 8.7) Occupation: Employees at 4 corporate worksites (public and private)	6 months	Overweight (BMI ≥ 23 kg/m^2^)	Multicomponent intervention to improve knowledge, attitude and health lifestyle, focused on healthy living, diet and physical activity. IG: 2 sessions/2 weeks on healthy living, diet, and PA + 2 PA training sessions + stress management sessions. CG: 2 general health talks in 6 months.	1. FBG, TC, HDL-c, LDL-c, TG. 2. Anthropometric measurements (WC, BMI, skinfold). 3. Other behavioural risk factors (tobacco and alcohol), dietary intake/food frequency, PA pattern.	IG achieved significant decrease in weight, BMI, WC, waist to hip ratio, skinfold (biceps, triceps, subscapular, suprailiac), FBG, TG; increase in HDL-c In CG, no significant difference in mean values except FBG and 3 skinfolds (biceps, triceps, subscapular). Weight loss in 12% of IG versus 4% of CG. (12.51 ± 10.38 cm vs. 3.50 ± 8.18 cm reduction in skinfold measurements). Greater conversion from sedentary to more active lifestyle in IG versus CG (67% to 55% vs. 69% to 65%). Lower calorie intake and fat consumption. More individuals in IG than in CG reduced number of risk factors for MetS.	Intervention achieved reduction in weight, subcutaneous fat and cardiometabolic risk factors after 6 months. The results could encourage other worksites in India to implement similar multicomponent interventions.
Proeschold-Bell et al., 2017 [30]	USA, IDF [36]	N: 1114 (IG1 395; IG2 283; IG3 436) M/F: 69.3%/30.7% Mean age: 51.9years (SD 10.0) Occupation: United Methodist clergy	24 months	No health status inclusion criteria	IG1: “Immediate intervention” and IG2: “1 year waitlist”: Personal goal setting + 3 workshops delivering stress management and theological content supporting healthy behaviours + 10–weeks online weight-loss program + small grant. IG3 “2-year waitlist: online stress management + journaling + exercises”.	1. MetS prevalence 2. Prevalence of depression, mean stress score, mean weight. Data collected at 0, 12, 18 and 24 months	Initial MetS prevalence: 50.9% Change in MetS prevalence in IG1: 49.5% to 42.9%; in IG2: 49.8% to 46.1%; in IG3: 49.6% to 45.1%. After 1 yr: 14% MetS prevalence (PR 0.86, 95% CI 0.79; 0.94, *p* < 0.001). After 2 years: −12% MetS prevalence sustained. Prevalence of components at baseline: central obesity 81.2%, low HDL 57.4%, hypertension 52.6%, high TG 50.9%, abnormal glucose regulation 13.7%. Beneficial effect at 24-months for the 3 most prevalent components Weight loss at 24 months: −3.4 kg for IG1; −4.4 kg for IG2; −1.7 kg for IG3. Depression and stress: no change in depression, gradual decrease in stress in all cohorts.	Spirited Life intervention improved MetS, central obesity, HDL, and BP over 24 months in US Christian Clergy. These findings support long-term behavioural change interventions.
Steinberg et al., 2015 [31]	USA NCEP-ATP-III [35]	N: 2835 (IG 1890; CG 945) M/F: 17%/83% Mean age: 46.53 years Occupation: Aetna employees who had previously participated in MetS biometric screening	12 months	≥2 MetS risk factors, one of which had to be WC	Personalised lifestyle-focused wellness programme. Contact with coaches and client care managers to achieve a healthier weight; focus on nutrition, PA, and behavioural well-being. Genetic profile for 3 genes associated with obesity, appetite and compulsory behaviour. IG1: given information about MetS results + invited to the program. IG2: MetS results + invited to the program + specific prediction of 12-mth future probability of MetS CG: given MetS results but not invited to program.	1. WC, TG, HDL-c, BP, FBG 2. Medical cost per employee-month during 12 months.	WC: greater reduction in IG2 vs. CG (−1.06 inches vs. −0.48 inches, *p* = 0.02). TG level: greater decrease in IG participants vs. CG (−18,47 mg/dL vs. −2.64 mg/dL, [*p* = 0.01]) Weight loss: 76% of IG participants lost weight; average weight loss 4.5 kg from baseline (*p* < 0.001). HDL-c: increase in IG1/IG2 vs. CG. Only IG2 showed significant increase (2.81 mg/dL vs. 1.44 mg/dL, *p* = 0.02). Total health care cost: lower in IG1/IG2 vs. CG ($312 vs. $434, *p* < 0.02).	A clinically targeted, personalised wellness program can significantly improve commitment and clinical outcomes related to MetS risk, as well as reducing costs, within just 1 year.
Kramer et al., 2015 [32]	USA NCEP-ATP-III [35]	N: 89 (IG 60; CG 29) M/F: 40/49 Mean age: 52.3 years (range 34–70) Occupation: Employees of Bayer Corporation	12 months	BMI ≥ 24 kg/m^2^ and evidence of prediabetes.	Lifestyle intervention to achieve and maintain 7% weight loss and to safely and progressively increase to 150 min/wk of moderate physical activity (e.g., brisk walking). IG: immediate intervention CG: delayed intervention (6 months after enrolment) All participants had weekly sessions for 12 weeks (face-to-face or DVD) and monthly meetings for 1 year. All participants were weighed at each in-person meeting and received handouts, pedometer, fat/calorie counter and exercise bands.	1. Change in weight at 6 months vs. baseline 2. BMI, BP, lipid profile, FBG, WC, HbA1c and PA. Data collected at baseline, 6, 12 and 18 months	Greater weight loss in IG vs. CG at 6 months (5.1% vs. 1%) as well as improved WC, HbA1, SBP/DBP, BMI and physical activity time. TG level decreased in IG but not in CG. Nonsignificant difference in TG decrease between groups. Higher proportion of IG achieved at least 5% or 7% weight loss vs. CG (45% vs. 7%, *p* = 0.0005, and 29% vs. 4%, *p* = 0.007). Nonsignificant difference in increase in weekly PA between IG and CG (75 min, IQR 30 to 126 vs. 40 min, IQR 0 to 112.5; *p* = 0.17). Increased HDL at 6 months in men and decrease in women. At 6 months (IG and CG): decreased weight (*p* < 0.001), HbA1c, SBP/DBP, WC, BMI; and increased PA. At 12 months (IG and CG): decreased TG, DBP/SBP, BMI, WC and HbA1c. At 18 months (IG and CG): decreased weight and WC; increased PA of 25 min/week with baseline (*p* = 0.04).	This intervention was effective in reducing weight and other risk factors for diabetes and CVD in this worksite setting
Puhkala et al., 2015 [33]	Finland, IDF [36]	N: 113 (IG 55; CG 58) M/F: NI Mean age: IG 47.6 years (SD 7.9) CG: 46.5 years (SD 8.6) Occupation: Long-distance bus and truck drivers.	24 months	WC ≥ 100 cm	Individual lifestyle counselling programme focused on improving nutrition, physical activity and sleep, to reduce body weight and MetS risk factors based on participants’ preferences, abilities and experience. IG: 12 months of counselling on diet, physical activity and sleep (6 individual 1-hr sessions and 7 30-min phone calls). Daily dietary, physical activity and sleep goals. CG: 3 months of counselling after 12 months (2 face-to-face sessions + 3 phone calls)	Weight, WC, glucose, TC, HDL, TG Questionnaire on health status and working conditions. Z-score to evaluate each MetS risk factor. Data collected at baseline, 12 months and 24 months.	Mean body weight change at 12 months: −3.4 kg (*p* = 0.001, range −26.1–9.9) kg in IG vs. +0.7 kg (*p* = 0.214, range −9.5–12.5) kg in CG. Net difference −4.0 kg (95% CI −6.2; −1.9) Weight loss at 12 months: 13% of IG lost ≥10% of initial body weight and 13% lost 5−9.9%. MetS prevalence at 12 months vs. baseline: 62% vs. 80% in IG and 60% vs. 62% in CG% (*p* = 0.34). Greater reduction in Z-score in IG vs. CG. MetS prevalence at 24 months: 60% in IG and 51% in CG. Non-significant difference between groups. Other results at 12 months: in IG, significant decrease in glucose and DBP; significant increase in HDL. Nonsignificant differences in LDL, TG, SBP. Z-score lower in IG.	The study showed clinically meaningful decreases in body weight and cardiometabolic risk factors after 12 months of counselling followed by 12 months of follow-up. Weight reduction and some improvement in cardiometabolic risk factors among long-distance truck and bus drivers is possible through lifestyle counselling, despite challenging working conditions.
Inoue et al., 2014 [34]	Japan NCEP-ATP-III [35]	N: 35 (IG 28; CG 7) M/F: 35/0 Mean age: 47.2 years (SD 7.9). Occupation: male office workers in the city hall	3 months	None partake in daily exercise	Japanese-style healthy lunch menu providing balanced nutrition and sufficient vegetables during 3 months (600–650 kcal, fat < 18 g, cholesterol ≤ 100 mg, fibre ≥ 8 g, total vegetables ≥ 130 g, sodium chloride equivalent ≤ 3.8 g). IG: received healthy lunch. For analysis, group divided into those who consumed healthy lunch < 50/61 times (< 50%ile) and ≥ 50/61 times (≥ 50%ile) CG: consumed usual lunches without restriction. Nutritional content was assessed. Weekend diet unrestricted for all participants.	TC, LDL, HDL, TG, HbA1c, glucose, leptin, anthropometric data and dietary intake.	CG at 3 months: no significant difference in anthropometrics data; increased SBP (*p* = 0.063). IG (< 50%ile) at 3 months: decreased DBP (*p* = 0.000). IG (≥ 50%ile) at 3 months: decreased SBP (*p* = 0.023), DBP (*p* = 0,001), TC (*p* = 0.006) and LDL (*p* = 0.010) Changes in nutritional intake: energy and carbohydrate intake significantly decreased in IG (< 50%ile); total dietary fibre and vegetables significantly increased in IG (≥ 50%ile).	Japanese-style healthy lunches (consumed consistently) decreased blood pressure and serum lipids and increased plasma ghrelin levels. Our study demonstrates that a short-term intervention consisting of Japanese-style healthy lunches at a workplace cafeteria contributes to lipid metabolism regulation.
Chen et al., 2013 [35]	Taiwan, MetS criteria of the Bureau of Health Promotion in Taiwan [37]	N: 63; (IG: 31 CG: 32) Mean age: CG 45.66 years (SD 8.32); IG: 41.90 years (SD 9.80) Occupation: Full-time career women	3 months	MetS risk factors.	Internet-based tailored health management platform. IG: 3-month intervention focused on nutrition, exercise recommendations and personal advice. CG: no intervention	Changes in health behaviour. MetS risk factors after 3 months (WC, TG, HDL, LDL, FBG, SBP/DBP)	Improvements for IG vs. CG at 3 months: WC (−3.5 vs. −0.6 cm, *p* < 0.05); fasting glucose (−6.5 vs. −3.1, *p* < 0.05); mean number of MetS risk factors (−0.6 vs. −0.011, *p* < 0.05). At 1.5 months, nutrition score had improved in both groups. At 3 months, both groups showed improvements in health behavioural score (*p* = 0.02), nutrition score (*p* = 0.02) and mental health score (*p* = 0.03).	A 3-month internet-based health intervention helped reduce participants’ waist circumference, fasting glucose and number of risk factors for MetS.
Allen et al., 2012 [36]	USA NCEP-ATP-III [35]	N: 64 (IG 26; CG 29) M/F: 6/58 Mean age: 48.9 years Occupation: Employees of the University of New Hampshire Cooperative Extension	12 months	No health status inclusion criteria	The workplace health promotion programme consisted of 10 monthly lifestyle education sessions delivered online and focused on health topics such as CHD risk, diabetes, importance of healthy diet and PA. IG: health risk screening + education sessions + pedometers. Interactive question-and-answer session and sampling of foods. Foods chosen for their nutritional benefit and ease of preparation; healthy snack options. CG: health risk screening + minimal information	1. Percentage-point reduction in LDL-C. 2. 10-year risk for CHD according to Framingham Risk Score (TC, HDL, LDL, glucose, CRP, BMI, SBP/DBP, body fat, cigarette smoking).	After 12 months, mean LDL-c (SD) was significantly lower in IG vs. CG (110.9 [22.2] mg/dL vs. 126.7 [21.8] mg/dL), with a relative difference between groups of 13.4%; no change in CG from baseline. Mean (SD) TC was significantly lower after 12 months in IG (183.4 [22.2] mg/dL) vs. CG (198.6 [20.9] mg/dL); no change in CG from baseline. WC increased in CG and unchanged in IG after 12 months. MetS markers: higher number of markers in CG vs. IG at 12 months (1.9 vs. 1.3). No difference between groups at 12 months in Framingham Risk Score, but absolute reduction of 0.3 points in CHD risk in IG (relative improvement of 18%). Number of steps increased from baseline to 12 months.	Compared with statin administration or lifestyle education in a clinical setting, intervention by videoconference is cost-effective and reduces LDL-c and overall CHD risk.
Nanri et al., 2012 [37]	Japan, Japanese definition of MetS [38]	N: 102 (IG 49; CG 53) M/F: 102/0 Mean age: 53.2 years (SD 6.8, range 38–68]. Occupation: Male employees of company in Japan	6 months	WC ≥ 85 cm plus ≥ 2 MetS risk factors	Lifestyle modification programme based on behavioural change theory. IG: Individual advice for health-related lifestyle changes, including diet and physical activity. Facilitators for behavioural change: pedometers, scales, leaflet and a diary to record behavioural performance and body changes. CG: standard guidelines	1. MetS prevalence at six months. 2. Changes in prevalence of abdominal obesity, dyslipidaemia, BP, hyperglycaemia; and mean change in MetS components (WC, weight, BMI, BP, TC, HDL-c, TG, glucose, HbA1c, CRP).	MetS prevalence did not differ significantly between the two groups (65.3% in IG vs. 62.3% in CG; *p* = 0,75). No significant differences were observed in BP, TC, HDL-c, TG or glucose. In the IG, intake of cereals and sugar/sweeteners significantly decreased. Rice decreased from 357 g to 297 g (*p* = 0.004) and PA increased by 57 min/wk (*p* < 0.001) after 6 months and no change in the control group (*p* = 0.99).	The intervention did not decrease MetS prevalence. Weight, WC and HbA1c were significantly lower in the IG vs. CG, and the IG made more healthy changes such as reducing sugar, cereals and sweets, and increasing physical activity.
Maruyama et al., 2010 [38]	Tokyo, Japan, NI	N: 87 (IG 52; CG 47) M/F: 87/0 Age range: 30 to 59 years Occupation: Office workers belonging to a health insurance association	4 months	MetS risk factors based on results of regular health check-ups.	Lifestyle modification programme to promote healthy dietary habits and PA. IG: 4 individual counselling sessions with a dietitian and a physical trainer. Participants registered their current targeted food intake and pedometer data on a website for self-monitoring, website advice and personal counselling from the counsellors. CG: no intervention	1. Food group intake and number of steps. Weight, WC, BP, BMI, TC, TG, HDL-c, LDL-c, HA1c, insulin.	Increased consumption of healthy food and decreased consumption of unhealthy food in IG (*p* = 0.00) but not in CG. Change in number of steps was similar in both groups. Significantly higher percentage of subjects with improvements in clinical parameters in IG compared with CG.	Generalised and relatively simple lifestyle changes, encouraged by a counsellor appear to help prevent metabolic disorders. Interventions based on personal contact and interactive resources are necessary to confirm long-term effects.
Racette et al., 2009 [39]	USA (NCEP-ATP-III) [35]	N: 151 M/F: 17/134 Mean age: 45 years (SD 9) Occupation: employees at selected worksites within a large medical centre	12 months	All employees were eligible.	Health promotion program based on behaviour change theory. IG: assessments + intervention on nutrition and PA + incentives to promote healthy behaviours + pedometers, weekly healthy snack cart, on-site WeightWatchers group meetings and group exercise classes, monthly lunchtime seminars and newsletters, walking team competitions and participation rewards CG: annual health assessment only	1. Weight, BMI, body composition, BP, fitness, lipids, Framingham risk score. 2. TC, HDL-c, LDL-c, TG, FBG. 3. Changes in food group intake and total daily PA	Both groups showed improvements in fitness, BP, HDL-c and LDL-c, and a slight reduction in weight, BMI and fat mass (greater reduction in IG). In the IG, the proportion of participants with the lowest Framingham Risk Score increased from 40% at baseline to 57% after 1 year. The prevalence of MetS reduced significantly in the IG from 38% to 25%, owing to improvements in HDL-c and BP. IG participants also increased fruit and vegetable intake from 4.7 servings at baseline to 7.8 servings at 6 months and 7.0 at 1 year (all *p* < 0.001); decreased consumption of saturated fat, fatty meals and fried foods (*p* < 0.001); and significantly increased total daily PA. CG showed significant but smaller improvements in fruit and vegetable intake, saturated fat intake and PA.	Multi-component worksite intervention achieved significant improvements in CVD risk factors and physical fitness. These benefits were attributable to the health assessments and personalized feedback rather than the intervention.

BMI: body mass index; BP: blood pressure; CHD: cardiovascular heart disease; DBP: diastolic blood pressure; FBG: fasting blood glucose; HbA1c: glycated haemoglobin; HDL-c: high-density lipoprotein cholesterol; IDF: International Diabetes Federation; LDL-c: low-density lipoprotein cholesterol; M/F: number of men/number of women; MetS: metabolic syndrome; PA: physical activity; SBP: systolic blood pressure; TC: total cholesterol; TG: triglycerides; WC: waist circumference; NI: nutritional intervention; GI: intervention group; GI1: intervention group 1; GI 2: intervention group 2; CG: control group.

**Table 2:** Assessment of Study Quality According to the 25-Item CONSORT Guidelines.

Study	Checklist Item																										
	1	2	3	4	5	6	7	8	9	10	11	12	13	14	15	16	17	18	19	20	21	22	23	24	25	Total	(%)
Woo et al., 2020 [27]	0.5	1	0.5	1	1	0.5	0	1	0	0	0.5	1	0.5	0.5	1	1	0	0	0	1	1	1	0	1	0	14	56
Kempf et al., 2019 [28]	1	1	0.5	1	1	0.5	0.5	1	1	1	0.5	1	1	0.5	1	1	0.5	0	1	1	1	1	1	1	1	20.5	82
Shrivastava et al., 2017 [29]	1	1	0.5	1	1	0.5	0.5	1	0	0	0	1	0.5	0	1	0	0	1	0	1	1	1	1	0	0	13.5	54
Proeschold-Bell et al., 2017 [30]	0.5	0.5	0.5	1	0	0.5	0.5	1	1	1	0.5	1	1	0	1	1	1	0	0	1	0	0	1	1	1	16	64
Steinberg et al., 2015 [31]	0.5	1	0	0.5	0	0.5	0	1	0	0	0	1	1	0.5	1	0	0	0	0	1	1	1	0	1	0	11.5	46
Kramer et al., 2015 [32]	0.5	1	0.5	1	0	0.5	0	0.5	0	0	0	0.5	0.5	0.5	1	1	0.5	0	0	1	1	1	0	1	0	12	48
Puhkala et al., 2015 [33]	1	1	0.5	05	1	0.5	0.5	1	1	1	1	1	0	0	1	1	0.5	1	0	1	1	1	0	1	1	19	76
Inoue et al., 2014 [34]	0.5	1	0.5	1	1	1	0	0	0	0	0	0.5	0.5	0	1	0	0	0	0	1	0	1	0	1	1	11	44
Chen et al., 2013 [35]	0.5	1	0.5	1	1	0	0	0.5	0	0	0	0.5	1	0.5	1	1	0	0	0	1	0	1	0	1	0	10	40
Allen et al., 2012 [36]	0.5	1	0	1	0	0.5	0	0.5	0	0	0	0.5	0.5	0	1	0	0.5	0	0	1	0	1	0	0	1	9.5	38
Nanri et al., 2012 [37]	1	1	0.5	1	0	0.5	0	0.5	0	0	0	0.5	0.5	0	1	1	0.5	0	0	1	0	1	1	1	1	12.5	50
Maruyama et al., 2010 [38]	1	1	0.5	1	1	0	0.5	0.5	1	1	0.5	0.5	1	0.5	1	0	0.5	0	0	1	0	1	0	1	1	15	60
Racette et al., 2009 [39]	0.5	1	0	1	1	0.5	0	0	0	1	0	1	1	0.5	1	1	1	0	1	1	0	1	0	1	1	16	64

Score 0: if not applicable; Score 0.5: if partially applicable; Score 1: if fully applicable. 1: Title/abstract; 2: Introduction; 3: Trial design; 4: Participants; 5: Interventions; 6: Outcomes; 7: Sample size; 8: Randomisation; 9: Allocation; 10: Implementation; 11: Blinding; 12: Statistical methods; 13: Participant flow; 14: Recruitment; 15: Baseline data; 16: Numbers analysed; 17: Outcomes and estimation; 18: ancillary analysis; 19: Hamms; 20: Limitations; 21: generalisability; 22: interpretation; 23: Registration; 24: Protocol; 25: Funding.

**Table 3:** Intervention Types.

	Intervention Led by	Int.1	Int.2	Int.3	Int.4	Int.5	**Int.6**
Woo et al., 2020 [27]	Health administrators, doctors and nutritionists	x	x	x	x		x
Kempf et al., 2019 [28]	Diabetes nurses trained in mental and motivational coaching			x	x		x
Shrivastava et al., 2017 [29]	Physicians, nutritionist and physical trainer	x			x	x	x
Proeschold-Bell et al., 2017 [30]	Intervention health coaches	x		x		x	x
Steinberg et al., 2015 [31]	Personal coaches and client care managers		x	x	x		x
Kramer et al., 2015 [32]	Trained prevention professionals as lifestyle coaches and a nurse practitioner	x	x		x		x
Puhkala al, 2015 [33]	Nutritionists and a physiotherapist	x	x		x	x	x
Inoue et al., 2014 [34]	Cook in staff cafeteria		x				
Chen et al., 2013 [35]	Health management expert		x		x		x
Allen et al., 2012 [36]	Lifestyle professionals	x		x			x
Nanri et al., 2012 [37]	Trained occupational health nurse	x		x	x		
Maruyama et al., 2010 [38]	Dietitian and physical trainer (certified health counsellors for the program)		x	x	x		x
Racette et al., 2009 [39]	Dietitian and exercise specialist		x	x	x		x

Int.1: basic education and general counselling on healthy living and diet; Int.2: specific diet/changes in diet and food intake; Int.3: behavioural changes/coaching; Int.4: physical exercise education and/or training; Int.5: stress and/or sleep management; Int.6: internet/social networks; x: the study included this type of intervention.

**Table 4:** Percentage Heterogeneity for Nine Different Parameters in Leave-One-Out Analysis (Random Effects Model).

ID	Omitting	WC	BMI	TC	HDL-c	LDL-c	TG	SBP	DBP	FBG
1	Woo et al., 2020	82.20%	95.51%	67.83%	52.43%	70.30%	65.68%	96.58%	89.62%	32.81%
2	Woo et al., 2020	81.96%	95.51%	61.00%	51.85%	59.79%	66.86%	96.59%	88.67%	37.11%
3	Woo et al., 2020	82.21%	95.49%	67.65%	50.46%	72.83%	65.54%	96.58%	89.64%	37.52%
4	Kempf et al., 2019		95.49%					94.98%	84.72%	
5	Kempf et al., 2019		95.49%					95.49%	89.05%	
6	Kempf et al., 2019		89.54%					96.59%	89.61%	
7	Shrivastava et al., 2017	80.80%	92.88%	53.80%	48.76%	68.21%	51.68%	96.38%	89.54%	33.25%
8	Shrivastava et al., 2017	82.17%	95.17%	68.08%	38.05%	71.26%	66.59%	96.25%	89.00%	10.40%
9	Kramer et al., 2015	80.98%	95.50%	66.77%	52.62%	72.83%	64.86%	95.57%	85.79%	37.50%
10	Kramer et al., 2015	80.40%	95.28%	67.83%	51.08%	72.77%	66.66%	96.51%	89.38%	34.15%
11	Kramer et al., 2015	78.81%	95.18%	68.08%	52.70%	72.35%	65.25%	96.57%	89.63%	29.95%
12	Kramer et al., 2015	78.45%	95.42%	66.50%	52.43%	71.35%	66.84%	96.59%	89.60%	30.59%
13	Puhkala et al., 2015	79.72%			52.23%					34.62%
14	Puhkala et al., 2015	78.38%			48.43%					29.32%
15	Inoue et al., 2014	82.22%	95.50%	68.07%	49.86%	72.84%	66.55%	96.59%	89.64%	37.60%
16	Inoue et al., 2014	82.17%	95.49%	67.17%	50.58%	72.22%	66.84%	96.59%	89.52%	35.61%
17	Chen et al., 2013	82.13%			44.97%		66.52%	96.59%	89.61%	35.42%
18	Allen et al., 2012	79.67%	95.51%	58.84%	48.47%	70.79%	66.82%	96.25%	89.50%	32.75%
19	Nanri et al., 2012		95.44%	65.24%	49.87%		52.21%	96.52%	87.71%	31.23%
20	Nanri et al., 2012	82.16%	95.51%	66.29%	52.65%		64.06%	96.55%	88.87%	37.30%
21	Maruyama et al., 2010	82.04%	95.49%	66.24%	51.09%	72.51%	60.19%	96.54%	89.57%	37.62%
22	Racette et al., 2009		95.51%	67.70%	43.75%	62.19%	65.11%	96.59%	89.31%	36.78%
	Pooled estimate	81.07%	95.25%	65.84%	49.93%	70.58%	64.67%	96.41%	89.06%	33.96%

ID: Identifier; WC: waist circumference; BMI: body mass index; TC: total cholesterol; HDL-c: high-density lipoprotein cholesterol; LDL-c: low-density lipoprotein cholesterol; TG: triglycerides; SBP: systolic blood pressure; DBP: diastolic blood pressure; FBG: fasting blood glucose.

**Table 5:** Number of Added Studies and Estimated Effect Size by Trim-and-Fill and Copas Methods.

		Trim-and-Fill			Copas		
		Random Effects Model			Random Effects Model		
	Variable	No. of Added Studies	Effect Size	95% CI	No. of Added Studies	Effect Size	95% CI
1	WC	0			0		
2	BMI	6	−1.10	[−1.45; −0.75]	0		
3	TC	5	−3.65	[−7.08; −0.23]	3	−5.00	[−7.38; −2.63]
4	HDL-c	3	1.08	[0.28; −1.88]	0		
5	LDL-c	6	−1.68	[−5.47; −2.10]	8	−2.03	[−4.01; −0.06]
6	TG	4	−5.79	[−13.13; 1.54]	0		
7	SBP	10	−8.24	[−10.84; −5.64]	0		
8	DBP	7	−4.67	[−5.78; −3.57]	0		
9	FBG	0			0		

WC: waist circumference; BMI: body mass index; TC: total cholesterol; HDL-c: high-density lipoprotein cholesterol; LDL-c: low-density lipoprotein cholesterol; TG: triglycerides; SBP: systolic blood pressure; DBP: diastolic blood pressure; FBG: fasting blood glucose.

**Table 6:** Moderator Analysis: Influence of Intervention Type and Duration on the Variables Studied.

Variable	Period	Sig.	Int.1	Sig.	Int.2	Sig.	Int.3	Sig.	Int.4	Sig.	Int.5	Sig.	Int.6	Sig.
WC	−0.03	0.898	1.29	0.170	0.33	0.751	−0.79	0.424	−2.19	0.035	−0.09	0.943	−0.99	0.346
BMI	−0.09	0.065	0.78	0.006	0.49	0.293	−0.79	0.009	−0.77	0.034	0.32	0.655	−0.51	0.139
TC	0.03	0.963	−3.37	0.323	−3.40	0.354	−3.07	0.372	0.90	0.803	2.41	0.696	1.38	0.688
HDL-c	0.06	0.548	−0.64	0.430	−0.37	0.660	1.58	0.043	2.02	0.015	0.25	0.784	1.21	0.136
LDL-c	0.18	0.738	−2.41	0.521	−6.42	0.063	−5.41	0.136	−1.57	0.695	5.50	0.371	−3.63	0.331
TG	0.3	0.784	−4.68	0.519	−1.23	0.874	−3.55	0.616	−0.84	0.914	1.09	0.930	6.01	0.418
SBP	−0.45	0.077	4.85	0.038	−0.91	0.758	0.72	0.799	−1.44	0.614	2.90	0.604	−3.53	0.175
DBP	−0.33	0.003	3.34	0.001	−2.25	0.087	0.50	0.670	0.38	0.754	0.61	0.777	−1.46	0.193
FBG	0.21	0.032	−1.26	0.071	−0.52	0.578	1.79	0.002	−0.09	0.925	−1.15	0.144	−0.65	0.460

WC: waist circumference; BMI: body mass index; TC: total cholesterol; HDL-c: high-density lipoprotein cholesterol; LDL-c: low-density lipoprotein cholesterol; TG: triglycerides; SBP: systolic blood pressure; DBP: diastolic blood pressure; FBG: fasting blood glucose. Int.1: basic education and general counselling on healthy living and diet; Int.2: specific diet/changes in diet and food intake; Int.3: behavioural changes/coaching; Int.4: physical exercise education and/or training; Int.5: stress and/or sleep management; Int.6: internet/social networks. Sig: statistical significance.

## References

[1] Saklayen M.G. (2018). The Global Epidemic of the Metabolic Syndrome. Curr. Hypertens. Rep..

[2] Alberti K.G., Eckel R.H., Grundy S.M., Zimmet P.Z., Cleeman J.I. (2009). International Diabetes Federation Task Force on Epidemiology and Prevention; National Heart, Lung, and BloodInstitute; American Heart Association; World Heart Federation; International Atherosclerosis Society; International Association for the Study of Obesity. Harmonizing the metabolic syndrome: A joint interimstatement. Circulation.

[3] Mottillo S., Filion K.B., Genest J., Joseph L., Pilote L., Poirier P., Rinfret S., Schiffrin E.L., Eisenberg M.J. (2010). Themetabolicsyndrome and cardiovascular risk: A systematic review and meta-analysis. J. Am. Coll. Cardiol..

[4] Grundy S.M. (2016). Metabolic syndrome update. Trends Cardiovasc. Med..

[5] Van Namen M., Prendergast L., Peiris C. (2019). Supervised lifestyle intervention for people with metabolic syndrome improves outcomes and reduces individual risk factors of metabolic syndrome: A systematic review and meta-analysis. Metabolism.

[6] Saboya P.P., Bodanese L.C., Zimmermann P.R., Da Silva Gustavo A., Macagnan F.E., Feoli A.P., Da Silva Oliveira M. (2017). Lifestyle Intervention on Metabolic Syndrome and its Impact on Quality of Life: A Randomized Controlled Trial. Arq. Bras. De Cardiol..

[7] Clifton P. (2019). Metabolic Syndrome—Role of Dietary Fat Type and Quantity. Nutrients.

[8] Sun K., Ren M., Liu D., Wang C., Yan L. (2014). Alcohol consumption and risk of metabolic syndrome: A meta-analysis of prospective studies. Clin. Nutr..

[9] Malik V.S., Popkin B.M., Bray G.A., Després J.-P., Willett W.C., Hu F.B. (2010). Sugar-Sweetened Beverages and Risk of Metabolic Syndrome and Type 2 Diabetes: A meta-analysis. Diabetes Care.

[10] Pérez-Martínez P., Mikhailidis D.P., Athyros V.G., Bullo M., Couture P., Covas M.I., de Koning L., Delgado-Lista J., Díaz-López A., Drevon C.A. (2017). Lifestyle recommendations for the prevention and management of metabolic syndrome: An international panel recommendation. Nutr. Rev..

[11] Burton W.N., Chen C.-Y., Schultz A.B., Edington D.W. (2008). The Prevalence of Metabolic Syndrome in an Employed Population and the Impact on Health and Productivity. J. Occup. Environ. Med..

[12] Schultz A.B., Edington D.W. (2009). The association between changes in metabolic syndrome and changes in cost in a workplace pop-ulation. J. Occup. Environ. Med..

[13] Schultz A.B., Edington D.W. (2010). Analysis of the Association between Metabolic Syndrome and Disease in a Workplace Population over Time. Value Health.

[14] Ryo M., Nakamura T., Funahashi T., Noguchi M., Kishida K., Okauchi Y., Nishizawa H., Ogawa T., Kojima S., Ohira T. (2011). Health Education “Hokenshido” Program Reduced Metabolic Syndrome in the Amagasaki Visceral Fat Study. Three-Year Follow-up Study of 3,174 Japanese Employees. Intern. Med..

[15] Mato V.V., Caddick N., King J.A., Johnson V., Edwardson C., Yates T., Stensel D.J., Daly H., Nimmo M.A., Clemes S.A. (2018). The Impact of a Novel Structured Health Intervention for Truckers (SHIFT) on Physical Activity and Cardiometabolic Risk Factors. J. Occup. Environ. Med..

[16] Marks S. (2016). Culturally Sensitive Education Can Decrease Hispanic Workers’ Risk of Metabolic Syndrome. Work. Health Saf..

[17] Wanden-Berghe C., Sanz-Valero J. (2012). Systematic reviews in nutrition: Standardized methodology. Br. J. Nutr..

[18] Pandis N., Chung B., Scherer R.W., Elbourne D., Altman D.G. (2017). CONSORT 2010 statement: Extension checklist for reporting within person randomised trials. BMJ.

[19] Cooper H., Hedges L.V., Cooper H., Hedges L.V., Valentine J.C. (2011). Reseach as a Scientific Process. The Handbook of Research Syntheses and Meta-Analysis.

[20] Baujat B., Mahé C., Pignon J.P., Hill C. (2002). A Graphical Method for Exploring Heterogeneity in Meta-Analyses: Application to a Meta-Analysis of 65 Trials. Stat. Med..

[21] Duval S., Tweedie R. (2000). Nonparametric Trim and Fill Method of Accouranging for Publication Bias in meta-analisys. J. Am. Stat. Assoc..

[22] Copas J.B., Shi J.Q. (2001). A Sensitivity Analysis for Publication Bias in Systematic Reviews. Stat. Methods Med. Res..

[23] Luo D., Wan X., Liu J., Tong T. (2018). Optimally estimating the sample mean from the sample size, median, mid-range, and/or mid-quartile range. Stat. Methods Med. Res..

[24] Wan X., Wang W., Liu J., Tong T. (2014). Estimating the sample mean and standard deviation from the sample size, median, range and/or interquartile range. BMC Med. Res. Methodol..

[25] Balduzzi S., Rücker G., Schwarzer G. (2019). How to perform a meta-analysis with R: A practical tutorial. Evid. Based Ment. Health.

[26] Schwarzer G., Carpenter J.R., Rucker G. (2021). Metasens: Statistical Methods for Sensitivity Analysis in Meta-Analysis, R Package Version 0.6-0. https://cran.r-project.org/package=metasens.

[27] Woo S.H., Oh E.G., Kim K.-S., Chu S.H., Kim G.S., Nam C.M. (2020). Development and Assessment of a Social Network Service-Based Lifestyle-Modification Program for Workers at High Risk of Developing Cardiovascular Disease. Work. Health Saf..

[28] Kempf K., Röhling M., Martin S., Schneider M. (2019). Telemedical coaching for weight loss in overweight employees: A three-armed randomised controlled trial. BMJ Open.

[29] Shrivastava U., Fatma M., Mohan S., Singh P., Misra A. (2017). Randomized Control Trial for Reduction of Body Weight, Body Fat Patterning, and Cardiometabolic Risk Factors in Overweight Worksite Employees in Delhi, India. J. Diabetes Res..

[30] Proeschold-Bell R.J., Turner E.L., Bennett G.G., Yao J., Li X.-F., Eagle D.E., Meyer R.A., Williams R.B., Swift R.Y., Moore H.E. (2017). A 2-Year Holistic Health and Stress Intervention: Results of an RCT in Clergy. Am. J. Prev. Med..

[31] Steinberg G., Scott A., Honcz J., Spettell C., Pradhan S. (2015). Reducing Metabolic Syndrome Risk Using a Personalized Wellness Program. J. Occup. Environ. Med..

[32] Kramer M.K., Molenaar D.M., Arena V., Venditti E.M., Meehan R.J., Miller R., Vanderwood K.K., Eaglehouse Y., Kriska A. (2015). Improving Employee Health: Evaluation of a worksite lifestyle change program to decrease risk factors for diabetes and car-diovascular disease. J. Occup. Environ. Med..

[33] Puhkala J., Kukkonen-Harjula K., Mansikkamäki K., Aittasalo M., Hublin C., Kärmeniemi P., Olkkonen S., Partinen M., Sallinen M., Tokola K. (2015). Lifestyle counseling to reduce body weight and cardiometabolic risk factors among truck and bus drivers – a randomized controlled trial. Scand. J. Work. Environ. Health.

[34] Inoue H., Sasaki R., Aiso I., Kuwano T. (2014). Short-term intake of a Japanese-style healthy lunch menu contributes to prevention and/or improvement in metabolic syndrome among middle-aged men: A non-randomized controlled trial. Lipids Health Dis..

[35] Chen Y.-C., Tsao L.-I., Huang C.-H., Yu Y.-Y., Liu I.-L., Jou H.-J. (2013). An Internet-based health management platform may effectively reduce the risk factors of metabolic syndrome among career women. Taiwan. J. Obstet. Gynecol..

[36] Allen J.C., Lewis J.B., Tagliaferro A.R. (2012). Cost-Effectiveness of Health Risk Reduction After Lifestyle Education in the Small Workplace. Prev. Chronic Dis..

[37] Nanri A., Tomita K., Matsushita Y., Ichikawa F., Yamamoto M., Nagafuchi Y., Kakumoto Y., Mizoue T. (2012). Effect of Six Months Lifestyle Intervention in Japanese Men with Metabolic Syndrome: Randomized Controlled Trial. J. Occup. Health.

[38] Maruyama C., Kimura M., Okumura H., Hayashi K., Arao T. (2010). Effect of a worksite-based intervention program on metabolic parameters in middle-aged male white-collar workers: A randomized controlled trial. Prev. Med..

[39] Racette S.B., Deusinger S.S., Inman C.L., Burlis T.L., Highstein G.R., Buskirk T.D., Steger-May K., Peterson L.R. (2009). Worksite Opportunities for Wellness (WOW): Effects on cardiovascular disease risk factors after 1 year. Prev. Med..

[40] National Cholesterol Education Program (2002). Third Report of the Expert Panel of Detection, Evaluation, and Treatment of High Blood Cholesterol in Adults.

[41] Alberti K.G.M.M., Zimmet P., Shaw J. (2006). Metabolic syndrome—A new world-wide definition. A Consensus Statement from the International Diabetes Federation. Diabet. Med..

[42] Hwang L.-C., Bai C.-H., Chen C.-J. (2006). Prevalence of Obesity and Metabolic Syndrome in Taiwan. J. Formos. Med. Assoc..

[43] Marzusawa Y. (2005). The Examination Committee of Criteria for “Metabolic Syndrome” in Japan. Criteria for “metabolicsyndrome” in Japan. J. Jpn. Soc. Int. Med..

[44] Harvard University (2011). School of Public Health. Healthy Eating Plate & Healthy Eating Pyramid. https://www.hsph.harvard.edu/nutritionsource/healthy-eating-plate/.

[45] Rosenstock I.M., Glanz K., Lewis F.M., Rimer B.K. (1990). The Health Belief Model: Explaining Health Behavior througt Expectancies. Health Behavior and Health Education: Theory, Research and Practice.

[46] Prochaska J.O., DiClemente C.C. (1983). Stages and processes of self-change of smoking: Toward an integrative model of change. J. Consult. Clin. Psychol..

[47] Williams R.B., Williams V.P. (2006). Control: No More Snapping at Your Family, Sulking at Work, Steaming in the Grocery Line, Seething in Meetings, Stuffifing your Frustration.

[48] Bodenheimer T. (2020). Coaching patients to be active, informed partners in their health. Fam. Syst. Health.

[49] Aracil-Lavado E., Wanden-Berghe C., Sanz-Valero J. (2017). Evaluation of quality of life according to the nutritional status of the adult palliative patient: Systematic review. Hosp. Domic..

[50] Bhui K., Dinos S., Galant-Miecznikowska M., De Jongh B., Stansfeld S. (2016). Perceptions of work stress causes and effective interventions in employees working in public, private and non-governmental organisations: A qualitative study. BJPsych Bull..

[51] Huang P.L. (2009). A comprehensive definition for metabolic syndrome. Dis. Model. Mech..

[52] Singh I.M., Shishehbor M.H., Ansell B.J. (2007). High-density lipoprotein as a therapeutic target: A systematic review. JAMA.

[53] Rahimi K., Bidel Z., Nazarzadeh M., Copland E., Canoy D., Ramakrishnan R., Pinho-Gomes A.-C., Woodward M., Adler A., Agodoa L. (2021). Pharmacological blood pressure lowering for primary and secondary prevention of cardiovascular disease across different levels of blood pressure: An individual participant-level data meta-analysis. Lancet.

[54] Han T., Richmond P., Avenell A., Lean M. (1997). Waist circumference reduction and cardiovascular benefits during weight loss in women. Int. J. Obes..

[55] Castro-Barquero S., Ruiz-León A.M., Sierra-Pérez M., Estruch R., Casas R. (2020). Dietary Strategies for Metabolic Syndrome: A Comprehensive Review. Nutrients.

[56] Huang J.-H., Li R.-H., Huang S.-L., Sia H.-K., Lee S.-S., Wang W.-H., Tang F.-C. (2017). Relationships between different types of physical activity and metabolic syndrome among Taiwanese workers. Sci. Rep..

[57] Haufe S., Kerling A., Protte G., Bayerle P., Stenner H.T., Rolff S., Sundermeier T., Kück M., Ensslen R., Nachbar L. (2019). Telemonitoring-supported exercise training, metabolic syndrome severity, and work ability in company employees: A randomised controlled trial. Lancet Public Health.

[58] Tsai H.H., Yeh C.Y., Su C.T., Chen C.J., Peng S.M., Chen R.Y. (2013). The effects of exercise program on burnout and metabolic syndrome components in banking and insurance workers. Ind. Health.

[59] Dahal P.K., Hosseinzadeh H. (2019). Association of health literacy and diabetes self-management: A systematic review. Aust. J. Prim. Health.

[60] Juvinyà-Canal D., Bertran-Noguer C., Suñer-Soler R. (2018). Alfabetización para la salud, más que información. Gac. Sanit..

[61] Thom D.H., Ghorob A., Hessler D., De Vore D., Chen E., Bodenheimer T.A. (2013). Impact of Peer Health Coaching on Glycemic Control in Low-Income Patients with Diabetes: A Randomized Controlled Trial. Ann. Fam. Med..

[62] Burgess E., Hassmén P., Welvaert M., Pumpa K. (2017). Behavioural treatment strategies improve adherence to lifestyle intervention programmes in adults with obesity: A systematic review and meta-analysis. Clin. Obes..

[63] Eftekhari S., Alipour F., Aminian O., Saraei M. (2021). The association between job stress and metabolic syndrome among medical university staff. J. Diabetes Metab. Disord..

[64] Yamaoka K., Tango T. (2012). Effects of lifestyle modification on metabolic syndrome: A systematic review and meta-analysis. BMC Med..

[65] Lin C.-H., Chiang S.-L., Tzeng W.-C., Chiang L.-C. (2014). Systematic Review of Impact of Lifestyle-Modification Programs on Metabolic Risks and Patient-Reported Outcomes in Adults with Metabolic Syndrome. Worldviews Evid. Based Nurs..

[66] Burton J., World Health Organization (2010). WHO Healthy Workplace Framework and Model: Background and Supporting Literature and Practices. https://apps.who.int/iris/handle/10665/113144.

[67] Pelletier K.R. (2011). A review and analysis of the clinical and cost-effectiveness studies of comprehensive health promotion and disease management programs at the worksite update VIII 2008 to 2010. J. Occup. Environ. Med..

[68] World Health Organization (2004). Global Strategy on Diet, Physical Activity and Health. https://apps.who.int/iris/handle/10665/43035.

[69] The European Programme FOOD (Fighting Obesity through Offer and Demand) (2011). Balance Nutrition at Work, Conference Report.

